# Advances in the Analytical Determination and Toxicological Assessment of Dithiocarbamates and Their Hydrolysis Products in Fruits, Vegetables, and Cereals: Methodological Evolution, Challenges, and Future Directions

**DOI:** 10.3390/toxics13100819

**Published:** 2025-09-26

**Authors:** Tommaso Pacini, Serenella Orsini, Emanuela Verdini, Elisa Cristofani, Alessandro Pelliccia, Stefano Sdogati, Claudio Colosio, Ivan Pecorelli

**Affiliations:** 1 Chemistry Department, Istituto Zooprofilattico Sperimentale dell’Umbria e delle Marche “Togo Rosati”, via G. Salvemini, 1, 06126 Perugia, Italyi.pecorelli@izsum.it (I.P.); 2 Postgraduate School of Professional Health, University of Milan, via Festa del Perdono, 7, 20122 Milano, Italy

**Keywords:** dithiocarbamates, analytical methods, food safety

## Abstract

Despite the widespread use of dithiocarbamate fungicides such as maneb, mancozeb, metiram, propineb, thiram, and ziram detected, according to EU legislation, via common degradation product carbon disulfide (CS_2_), recent and comprehensive reviews on analytical methods for their determination in plant-based foods are lacking. Given the well-documented toxicity shown by the experimental model for these pesticides, including neurotoxicity and endocrine disruption, harmonized and reliable analytical protocols are crucial for food safety monitoring and regulatory compliance. Dithiocarbamates, beyond CS_2_ release, have been associated with immunotoxicity, thyroid dysfunction, and potential carcinogenicity, raising further concern regarding chronic dietary exposure. Their metabolites may disrupt enzymatic activity and oxidative balance, enhancing systemic toxicity. Early methods, had limited sensitivity, poor reproducibility, and relied on hazardous solvents, reducing practical value. Although later advancements improved detection limits, modern procedures, including those proposed by the European Union Reference Laboratory (EURL), still show limitations. The EURL-recommended protocol involves acid hydrolysis using concentrated HCl, extraction with isooctane, heating to 85 °C, and rapid ice-bath cooling, which poses environmental concerns. Recovery efficiency remains inconsistent in some cases, and reproducibility within commodity groups is poor. This review discusses the status of methods for determining dithiocarbamates as individual compounds and via CS_2_ moiety.

## 1. Introduction

**Figure 1:**
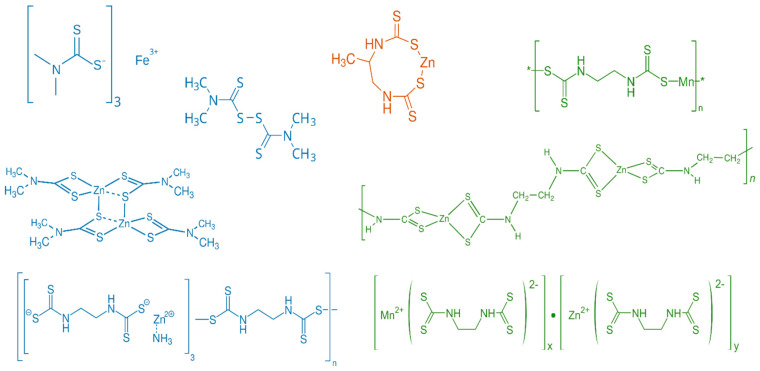
Chemical structure of different fungicide (dithiocarbamates): DMDCs (blue), from top to bottom, Ferbam, Thiram, Ziram, and Metiram; PBDCs (orange), Propineb; EBDCs (green), from (**top**) to (**bottom**) Maneb, Zineb, and Mancozeb.

Dithiocarbamates (DTCs) are synthetic organosulfur compounds allocated among the classes of fungicides most widely used in agriculture, mainly due to their cost-effectiveness and broad-spectrum activity. Compounds such as mancozeb, maneb, thiram, ziram, and propineb are routinely applied to fruits and vegetables to control fungal pathogens. However, their intensive application has raised serious concerns due to the persistence of residues in food commodities and the consequent risks for humans and the environment. Due to their chemical structure, DTCs can be allocated in three different categories: Dimethyl-dithiocarbamates (DMDCs), Ethylene-bis-dithiocarbamates (EBDCs), and Propylene-bis-dithiocarbamates (PBDCs) (Figure 1) [1].

**Figure 2:**
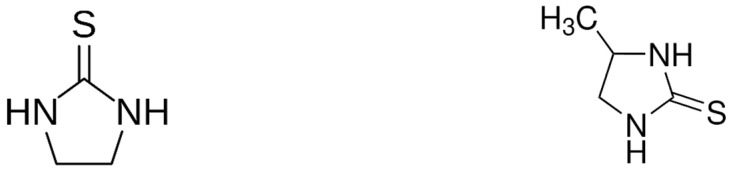
Ethylene thiourea (ETU) and propylene thiourea (PTU), toxic metabolites of EBDCs and PBDCs, respectively.

Ferbam, thiram, and ziram are examples of DMDCs. Ferbam and ziram are metal salts, iron, and zinc, respectively, of the dimethyldithiocarbamate anion, while thiram is an oxidized disulfide dimer of DMDC and does not contain any metal ions. These compounds are more soluble in organic solvents compared to other DTCs. Propineb, the sole PBDC, is a zinc salt of propylenebisdithiocarbamate, structurally distinguished by a propylene bridge linking two dithiocarbamate groups, which contributes to its polymeric nature and limited solubility [1]. EBDCs, including maneb, zineb, and mancozeb, are metal salts of ethylenebisdithiocarbamate, with maneb and zineb as the manganese and zinc salts, respectively, and mancozeb, which chelates a mixture of both metal ions. EBDCs and PBDCs are typically polymeric, poorly soluble, and degrade into more toxic metabolites like ethylene thiourea (ETU) and propylene thiourea (PTU), which have significant toxicological concerns (Figure 2). These structural and chemical differences affect not only their application and persistence in the environment but also the analytical approaches needed to detect them and assess dietary exposure risks [2].

Dithiocarbamates have been developed since 1931, when a first patent, claiming several disinfectants useful as bactericides, microbicide, and fungicide, including thiram (Dimethylcarbamothioic dithioperoxyanhydride) among others, was filed in the USA [3]. In 1934, the patent was granted, leading to the widespread production and commercialization, and the subsequent development, of these products for protecting crops. After the successful commercialization of thiram, due to its high efficiency and low toxicity, many other dithiocarbamates have been developed and commercialized as fungicides, such as the EBDCs, Maneb and Zineb, and the SMDCs, Ferbam and Ziram, in 1976 [4,5].

In 1977, the use of Propineb as a fungicide was first evaluated by the Joint FAO/WHO Meeting on Pesticide Residues (JMPR), and after further evaluations conducted in 1984, 1985, and 1993, it was finally approved in 2004. Its approval, however, was not renewed in 2018, causing the withdrawal of this compound from the list of active ingredients authorized in Europe [6]. As of today, other previously authorized DTCs have been phased out by the European Commission, even though they are still present in the MRL specification for dithiocarbamates, which includes Maneb, Mancozeb, Metiram, Thiram, Propineb, and Ziram in the legal complex definition. Among these fungicides, only Ziram is still approved in the EU, with an approval expiry date of 31 January 2027; however, even this dithiocarbamate is as of today a candidate for substitution [7]. Maneb was withdrawn in 2017 because no supplementary dossiers were submitted to support its renewal [8]. The authorization of Mancozeb was not renewed in 2020, with a grace period for use until January 2022 [9]. Metiram authorization was not renewed in 2023, with the possibility to use the existing stocks of the product until November 2024 [10]. Production, use, and sale of seeds treated with thiram, were finally prohibited in 2018 following the non-renewal of the approval for this chemical [11].

**Figure 3:**
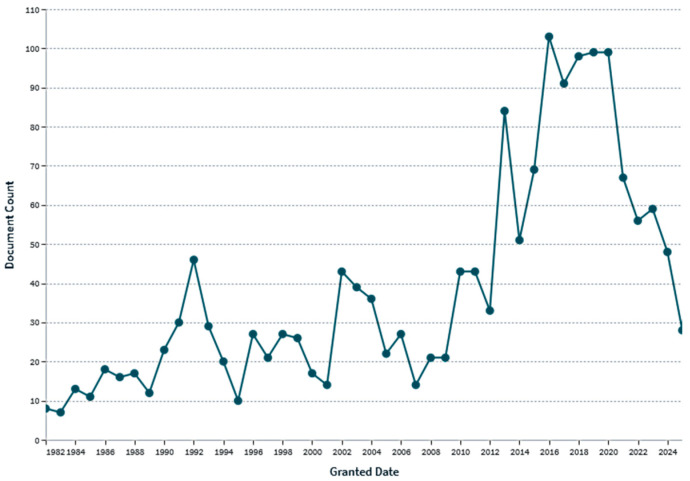
Patent trend of dithiocarbamates use as fungicides over the years in Europe (Lens.Org).

The MRL established by the EU refers to the levels of carbon disulfide (CS_2_) developed through the dithiocarbamates degradation, so it is impossible to attribute the CS_2_ levels developed to one fungicide or another. Moreover, the EU maintains a tolerance level for CS_2_, which could be generated by different fungicides, and continuously reviews and updates MRLs to reflect new scientific knowledge changes in pesticide authorizations, and alerts concerning the presence of pesticide residues in food products, taking into due account the principles of prevention and international trade considerations. The effect of the recent bans on different dithiocarbamates in Europe is evident through the trend of the volume of patent registrations in Europe linked to the two keywords “fungicide” and “dithiocarbamates” over time (Figure 3).

The patent trend shows that the peak of registrations was observed in 2015, with a constant growth since 2007, and production remained stable until 2020. In this period, novel formulations, combinations, and application methods have been developed, improving the stability of DTCs, enhancing their efficacy through synergistic mixtures with other active ingredients (e.g., dimethomorph and fosetyl-aluminum), reducing their environmental impact (e.g., improved dust-free formulations), and exploring new delivery systems [12,13,14,15].

The European Union’s commitment to safeguarding public health and environmental integrity is thus to maintain its rigorous approach to defining and updating the Maximum Residue Levels (MRLs) for dithiocarbamate fungicides. While the period between 2005 and 2015 witnessed a surge in the commercialization and patenting of new dithiocarbamate formulations, reflecting efforts to enhance efficacy and extend product life, this trend has been met with an increasingly stringent regulatory landscape. The subsequent phase-out of several key dithiocarbamates, including Propineb, Maneb, Mancozeb, Metiram, and thiram, is a direct consequence of the EU’s hazard-based assessment framework, in which the substances posing unacceptable risks to human and environmental health registration was withdrawn. Despite these withdrawals, the continued presence and dynamic adjustment of MRLs, expressed as the common analytical moiety CS_2_, remain critically important [16].

This ongoing regulatory activity serves multiple vital purposes: it addresses the complexities of analytical detection, manages legacy residues in the environment, and crucially navigates the intricacies of international trade by setting import tolerances for produce from non-EU countries where these substances may still be legally used. Furthermore, the evolving understanding of naturally occurring CS_2_ in some crops, e.g., *Brassica*, necessitates continuous MRL updates to ensure accurate monitoring and fair trade [17]. Thus, the EU Commission’s persistent efforts to set and update MRLs are not merely administrative tasks. They represent a fundamental pillar of consumer protection, a commitment to environmental stewardship, and a strategic response to the globalized nature of food supply, ensuring that only food deemed safe for all European consumers reaches their tables, regardless of its origin. This dynamic regulatory process is essential for maintaining the integrity of the EU food chain in an ever-changing agricultural and chemical landscape.

While earlier reviews such as that by Malik and Faubel in 1999 and Crnogorac and Schwack in 2008 provided an extensive overview of classical methods, including spectrophotometric, chromatographic, and polarographic techniques, subsequent methodological advances, particularly after 1999, have aimed to address limitations related to sensitivity, matrix effects, and reproducibility [18,19]. Despite these developments, several challenges persist, particularly in achieving selectivity among different DTCs using CS_2_-based indirect analysis, avoiding matrix-induced overestimation, and ensuring safety and environmental compatibility of analytical procedures.

This review aims to critically assess the evolution of analytical methods for the determination of dithiocarbamates in fruits and vegetables since 1999, with a particular emphasis on indirect CS_2_-based detection, in line with current EU legislation. Key developments in sample preparation, instrumental techniques, validation approaches, and matrix-specific adjustments will be evaluated, highlighting both advancements and existing methodological gaps.

## 2. Material and Methods

For the present literature review, references were retrieved from the Web of Science and Scopus databases. The selection was limited to research articles, patents, public institutions dithiocarbamates monitoring reports, and review papers published in peer-reviewed journals. Reports and publications from the European Food Safety Authority (EFSA), World Health Organization (WHO), International Agency for Research on Cancer (IARC), U.S. Environmental Protection Agency (EPA), and regulations of European Commission were also included in our consultation. For the patent analysis, two different databases were considered, Lens.org and the World Patent Office Database (WIPO), using the keyword “dithiocarbamate” for the research and removing the redundant results due to plural family members. Non-relevant patents have been removed manually. The search for non-patent research on analytical method collection focused on analytical methods specifically used and developed in the last 25 years (from 2000 to 2025) for the detection of dithiocarbamate in vegetables, fruit, and/or cereals to enable a follow-up on the evolution and trends over this period. Titles, abstracts, and keywords were manually screened to eliminate non-relevant publications.

In addition to the broader 25-year collection, particular attention was given to analytical methods documented in the primary literature over the last 5 years. For the determination of total dithiocarbamates via CS_2_ release, recent studies have explored a variety of instrumental approaches. Optical techniques, such as in situ Vis–NIR reflectance, have been investigated for direct monitoring [20], while chromatographic methods, including GC–MS, GC–ECD, and GC coupled to advanced detectors (ITD-MS, PFPD), have been widely applied [21,22,23,24,25]. More recently, innovative alternatives such as LC coupled with liquid electrode glow discharge–dielectric barrier discharge molecular emission spectrometry (LEGD–DBD–MES) and HPLC–MS/MS have been introduced, expanding the sensitivity and selectivity of CS_2_-based determination [26,27]. In parallel, research on the direct quantification of individual dithiocarbamates, without the need for CS_2_ release, has gained increasing attention. Spectroscopic methods such as UV–Vis, Raman, and surface-enhanced Raman spectroscopy have been frequently applied, often in combination with nanomaterials (e.g., gold or silver nanoparticles) to improve detection limits [28,29,30,31]. Fluorescence-based probes, including ratiometric and dual-mode colorimetric/fluorescent systems, have also been developed for specific analytes [32,33]. Chromatographic strategies remain highly relevant, with recent advances in HPLC coupled to tandem MS, ICP–MS, or novel detectors enhancing the capability to quantify multiple dithiocarbamates and their metabolites with high accuracy [34,35]. Collectively, these developments illustrate a diversification of methodologies, with both advanced chromatographic platforms and innovative spectroscopic/nanomaterial-based techniques driving the evolution of dithiocarbamate analysis in the most recent literature.

## 3. Dithiocarbamates Toxicological Profile

Dithiocarbamates are organosulfur compounds characterized by their ability to chelate different metal ions, including them in their structure as coordination atom (Zn, Fe, Mn, Na), except for Thiram. Although coordination with these metal ions is crucial for the efficiency of dithiocarbamate, in the last 10 years many concerns regarding their toxicological profiles have led to increasing scrutiny, particularly in relation to their long-term health effects and environmental persistence [36].

Thiram has been used extensively as a fungicide in seed treatment to control *fungi* that cause damping-off in seedlings and to control seedling blights and in foliar treatment on different fruits, vegetables, and ornamentals to control *Botrytis* species, rust, scab, and storage diseases [37]. It displays moderate acute toxicity in mammals, with oral LD_50_ values in rats ranging between 210 and 2000 mg/kg [38]. Dermal exposure can result in local irritation and sensitization. In terms of dietary exposure to crops contaminated with thiram, it is important to focus on sub-chronic and chronic studies, which have shown frequent hepatic and renal alteration as well as potential neurotoxicity [39]. Rats fed 52 to 67 mg/kg/day for 80 weeks exhibited hair loss, paralysis, and atrophy, while symptoms of muscle incoordination and paralysis from thiram poisoning have been shown to be associated with the degeneration of nerves in the lower lumbar and pelvic regions [40]. Repeated or prolonged exposure to thiram can cause allergic reactions such as dermatitis, watery eyes, sensitivity to light, and conjunctivitis [38]. Thiram inhibits aldehyde dehydrogenase, leading to a disulfiram-like effect in humans who consume alcohol after exposure, a reaction characterized by nausea, vomiting, and cardiovascular disturbances [41]. In developmental studies, high doses of thiram produced delayed ossification and other fetal abnormalities in rodents, although these effects were generally secondary to maternal toxicity [42].

Metiram is a zinc-containing EBDC fungicide structurally related to mancozeb and zineb. It exhibits low acute toxicity, with oral LD_50_ values in rats exceeding 6000 mg/kg [43]. However, its toxicological concern arises primarily from its degradation product, ethylene thiourea (ETU) (see ETU paragraph). In animal studies, chronic exposure to Metiram has resulted in thyroid enlargement, disrupted thyroid hormone homeostasis, and developmental abnormalities such as delayed ossification and reduced fetal weight. Though less extensively studied than maneb or mancozeb, Metiram’s shared metabolic pathway and toxic breakdown products indicate a similar risk profile, particularly regarding endocrine and developmental effects [42].

Zineb, a zinc-based EBDC, has low acute toxicity, with oral LD_50_ values in mammals between 1850 and 8900 mg/kg. However, sub-chronic exposure has been associated with histopathological changes in the thyroid gland and alterations in serum thyroid hormone levels, likely due to its interference with iodine uptake [44]. Also, Zineb is also a source of ethylene thiourea (ETU). Chronic feeding studies in rodents have suggested potential for thyroid and liver tumors, although the data are not still sufficiently robust for definitive carcinogenic classification [45].

Ziram, a zinc dimethyl dithiocarbamate, shares many toxicological features with thiram. It has a lower oral LD_50_, typically in the range of 400–480 mg/kg in mice and rabbits and causes severe eye and skin irritation upon contact [46]. Of growing toxicological interest is ziram’s potential neurotoxicity, characterized by its potential implication in dopaminergic neuron toxicity, possibly by interfering with ubiquitin-proteasome pathways and mitochondrial function, mechanisms that are also implicated in Parkinson’s disease [47]. Chronic exposure in animals has resulted in testicular atrophy, hepatic necrosis, and thyroid hyperplasia, suggesting that multiple organ systems are susceptible to its toxic effects [48].

Ferbam, a ferric dithiocarbamate, has a relatively low acute toxicity profile, but repeated exposure in animals is associated with hematological effects, particularly hemolytic anemia, and thyroid gland disturbances. Like other DTCs, its chronic toxic effects are partly attributable to ETU formation. Although some animal studies have shown increased incidence of thyroid tumors after prolonged exposure to ferbam, evidence of direct genotoxicity is lacking [42]. Ferbam can also induce skin sensitization in exposed workers, raising occupational safety concerns [49].

Propineb, structurally similar to zineb but based on a propylene diamine backbone, is commonly used on solanaceous crops. It has low acute toxicity, with oral LD_50_ values exceeding 5000 mg/kg in rats. This compound exhibits, in the experimental model, developmental toxicity at high doses, including skeletal abnormalities and reduced fetal body weight [50]. It also affects thyroid hormone levels and can induce hypertrophy of the thyroid gland. Chronic exposure studies indicate that the liver and hematopoietic system are additional targets of toxicity [51]. Propineb is of concern not only for its own toxicity but also because it is a source of PTU. The European Food Safety Authority’s peer-review of propineb shows that the active substance degrades extensively under hydrolytic conditions, forming PTU as a major product which causes, in the experimental model, endocrine-mediated adverse effects on the thyroid [51].

Mancozeb is a coordination product of zinc and manganese with ethylene bis(dithiocarbamate). While mancozeb’s acute toxicity is low, its chronic toxicity profile is more concerning. Repeated exposure in laboratory animals has led to thyroid follicular cell hyperplasia and neoplasia [52]. It is also a significant source of ETU, and there is also evidence that mancozeb could impair reproductive function, alter neurotransmitter levels, and affect neurodevelopment in animals [41].

Maneb, a manganese-based EBDC similar to mancozeb, has been extensively used in the cultivation of bananas, potatoes, and other crops. Like mancozeb, it degrades to ETU, thereby sharing its thyroid-disruptive properties. In developmental studies, maneb exposure was associated with delayed ossification, low fetal weights, and other signs of embryotoxicity [53]. Co-exposure to maneb and paraquat has been shown to produce dopaminergic neurodegeneration in animal models, mimicking features of Parkinson’s disease [54].

Overall, while acute toxicity of dithiocarbamates is generally low, chronic exposure studies show a range of risks, especially involving the thyroid gland, liver, reproductive system, and central nervous system. The common metabolite ETU represents a significant toxicological concern: ETU is primarily a metabolite of ethylene-bis-DTCs and can be present also as metabolite of other DTCs. For this reason, regulatory assessments of DTC fungicides often focus as much on ETU than on the parent compounds themselves. Its main toxicological effect is the inhibition of thyroid hormone synthesis, which induces a compensatory increase in TSH and can lead to goiter. This effect has been reported in the literature only once, in Bulgarian workers [55]. Persistent TSH stimulation may also increase the risk of thyroid neoplasms. Consequently, ETU was initially classified as Group 2B by IARC [56]. In 2001, however, it was reclassified into Group 3 (not classifiable as to its carcinogenicity to humans). The reclassification was based on the absence of evidence for genotoxicity, indicating a non-genotoxic mechanism of carcinogenesis, and on the fact that adverse effects were observed only in rodents at high experimental doses [45]. Concerns remain regarding teratogenic effects, observed in rodents at particularly high doses. Similar effects have been attributed to PTU, again at exposure levels unlikely to occur under typical occupational conditions or through dietary intake of residues.

Summarizing the data specifically discussed in the present paragraph, DTCs generally exhibit low acute toxicity, chronic and long-term effects, with particular focus on their metabolites: ETU, PTU, and CS_2_. Carbon disulfide is produced as a metabolite by nearly all DTCs; however, the conversion, ranging from 10 to 40% of the absorbed dose, makes the occurrence of the toxic effects in exposed populations highly unlikely [42].

ETU is primarily a metabolite of EBDTCs. Its main toxicological effect is the inhibition of thyroid hormone synthesis, which induces a compensatory increase in TSH and can lead to goiter. This effect has been reported in the literature only once, in Bulgarian worker [55]. Persistent TSH stimulation may also increase the risk of thyroid neoplasm. ETU is of concern also due to its teratogenic effects, observed in rodents in particularly high doses. Similar effects have been attributed to PTU, even if at exposure levels are unlikely to occur under typical occupational conditions or through dietary intakes of residues. A summary of the toxicological profiles of some DTCs and their main metabolites is provided in Table 1.

## 4. Analytical Methods for Dithiocarbamates

Dithiocarbamates are well known to degrade into carbon disulfide (CS_2_) under acidic conditions, a volatile compound that serves as the primary marker for their detection in regulatory monitoring. The toxicological relevance of CS_2_ and its parent compounds has prompted strict regulatory controls, particularly within the European Union. Under Commission Regulation (EU) 2017/171, the determination of DTC residues is mandated as a summation expressed in terms of CS_2_, encompassing various active substances, including mancozeb, maneb, metiram, propineb, thiram, and ziram [59]. This regulatory requirement has shifted analytical efforts toward robust, harmonized methods that prioritize the reliable quantification of CS_2_ as a proxy for the total dithiocarbamate content.

Over the last decade, two recent reviews, by Chung in 2022 and Campanale in 2023, have provided valuable contributions to the current understanding of DTCs, particularly in relation to their environmental fate, toxicological relevance, and analytical challenges. However, since the publication of those reviews, several key developments, both in regulatory methodology and analytical technique, have occurred, which now necessitate an updated, more comprehensive synthesis of available data and techniques [2,60]. One of the most critical advances has been the revision of the official EU Single Residue Method (SRM) for DTCs by the European Union Reference Laboratory (EURL). Between late 2023 and 2024, this method underwent several updates (Versions 3.0 through 3.2), introducing important changes to cleavage conditions, GC-MS/MS parameters, hydrolysis reagent composition, and calibration standards. These updates are central to the analytical detection of DTCs in plant-based foods and directly affect the performance, specificity, and comparability of results across laboratories [61].

Chung, in 2022, recognizes the fundamental limitation of CS_2_-based indirect methods, which is their inability to distinguish between specific DTC compounds; this recognition is, however, not followed by a systematic evaluation of recovery rates, limits of quantification (LOQs), or matrix-dependent variability. Similarly, Campanale, in 2023, offers an overview of DTC properties and environmental toxicity but does not substantively engage with the analytical challenges posed by individual compounds such as ziram, metiram, and propineb, which, even under revised EURL methods, still demonstrate recoveries equal to or lower than 50% [2,60,61].

Another critical shortcoming of the earlier literature is the continued reliance on hot acid digestion methods for CS_2_ determination, first developed by Clarke et al. in the early 1950s [62], despite well-documented issues with false positives in certain plant matrices (e.g., Brassica species and fruits like papaya), which naturally contain sulfur-containing compounds that yield CS_2_ under acid hydrolysis [63]. While Chung touches on this issue and briefly mentions alternative chromatographic approaches for individual DTCs, there is no holistic comparison of direct vs. indirect methods that consider both analytical performance and toxicological relevance. Moreover, the need for a more comprehensive analytical approach for ethylene thiourea (ETU) is critical due to its toxicological properties. In 2021, the U.S. National Toxicology Program reclassified ETU as a “Reasonably Anticipated Human Carcinogen.” This reclassification, along with ongoing reassessments of acceptable daily intakes (ADIs) for DTCs and their metabolites by the European Food Safety Authority (EFSA), highlights the urgent need for reliable, compound-specific residue monitoring [64].

Veiga-del-Bano et al., in 2023, conducted a bibliometric analysis of 374 publications on dithiocarbamate fungicides in the food sector (2012–2021), identified a 32% decline in scientific output over the last two years, spotlighted leading journals, countries, and institutions, and highlighted emerging research trends, such as focusing on specific fungicides (like mancozeb, thiram, and maneb), their metabolites, and a shift toward HPLC over traditional GC-based analytical methods [65]. In 2025, Martins et al. finally presented a valuable overview of carbamate and dithiocarbamate analysis in food. The review is broad in scope and not specific to dithiocarbamates, with limited critical evaluation of emerging techniques [66].

However, a significant gap remains: the connection between these toxicological findings and method validation data is not fully explored, leaving a fragmented picture for regulators and laboratories. A more complete analytical approach is necessary to bridge this gap and provide a clearer understanding of the risks associated with ETU.

The present review aims to provide a comprehensive, methodologically integrated, and practically oriented update on the last twenty-five years’ analysis of dithiocarbamates in plant-based food, incorporating and critically assessing the changes introduced in EURL-SRM versions 3.0 to 3.2, providing a comparative analysis of performance parameters (recovery, LOQ, matrix effects) for both indirect CS_2_-based methods and direct detection of single DTC compounds, and discussing the specific challenges associated with compounds like ziram, metiram, and propineb, which remain problematic despite method revisions.

### 4.1. Analytical Methods Involving the Development of CS_2_

Since the earliest investigations into DTCs residues, dating as far back as the 1940s, analytical methodologies have predominantly relied on the indirect determination of DTCs through the measurement of carbon disulfide (CS_2_) generated by acid hydrolysis. This approach rapidly became the standard due to its relative simplicity and its suitability for detecting a broad range of structurally diverse DTCs as a collective class. Even today, this methodology remains the reference approach in most regulatory frameworks, including the European Union, where Commission Regulation (EU) 2017/171 of January 2017 explicitly stipulates the quantification of DTC residues as the sum of CS_2_ (developed after breaking down of Maneb, Mancozeb, Metiram, Propineb, Thiram, and Ziram) released under specific acidic hydrolysis conditions [59]. The standardization around CS_2_ development has thus shaped the landscape of DTC analytical chemistry for over half a century.

Historically, and especially prior to 2005, the most widely adopted analytical protocols were based on modifications of the Cullen method, a foundational technique involving the production of CS_2_ under strongly acidic conditions and high temperature, the subsequent adsorption into an ethanol solution, and the colorimetric detection after the formation of a yellow chelate through the reaction of CS_2_ developed with Cu^2+^ and an alkylamine. The process is usually conducted in a horizontal system with an in-series double trap in order to improve efficiency and safety [67]. This method saw widespread application in routine monitoring, often in modified forms promoted by Keppel, who first introduced a reducing agent, SnCl_2_, before the treatment with hot acid, and a lead acetate solution to remove H_2_S and other interference, increasing the recovery of the DTC; and Caldas adaptations, by a vertical distillation system which offered operational flexibility and improved safety and reproducibility [68,69]. These protocols were well suited for batch processing and accommodated the relatively low technical sophistication available in early residue laboratories.

Detection of CS_2_ in this period typically relied on colorimetric techniques, particularly UV-Vis spectrophotometry, following derivatization [17,70,71,72,73,74,75]. While cost-effective and simple, these methods were affected by low specificity, limited sensitivity, and high susceptibility to matrix interferences, making them less reliable for trace analysis or complex sample matrices. They also often lacked rigorous calibration standards, which led to challenges in quantifying low-level residues accurately, especially near maximum residue limits (MRLs) [76,77].

In the years following 2005, analytical capabilities improved significantly with the increasing accessibility and routine use of gas chromatography coupled with mass spectrometry (GC-MS). This shift enabled greater sensitivity, selectivity, and quantification accuracy, allowing for robust identification and confirmation of CS_2_, even in trace concentrations, with limit of qualification (LOQ) lower than 0.05 mg/kg in some cases [78,79].

In addition to MS, other detectors such as flame photometric detectors (FPD) and electron capture detectors (ECD) have also been utilized for CS_2_, particularly in cases where instrumentation constraints or cost considerations limited MS deployment [80]. These newer GC-based methods represented a significant advance over classical colorimetric protocols, enhancing both analytical confidence and regulatory compliance.

Despite the dominance of GC-based techniques, a limited number of methods have explored liquid chromatography (LC or HPLC) for CS_2_ detection. The earlier literature described analytical methods for carbon disulfide determination based on derivatization with 1,2-benzenedithiol to form 1,3-benzodithiole-2-thione, followed by separation and quantification using reversed-phase liquid chromatography with UV detection [81]. This cyclocondensation approach, developed in the late 20th century, was widely applied for thiocarbonyl and dithiocarbamate analysis due to the stability and strong chromophore of the derivative. However, no updated or validated applications of this derivatization-HPLC method for direct CS_2_ measurement have been reported in the scientific literature since the early 2000s, and in the last two decades, this approach has remained uncommon.

A few niche methods, finally, have attempted detection via DBS-MES detectors or IR, yet these remain isolated and have not entered routine regulatory or monitoring use [82,83].

While the analytical determination of dithiocarbamates through the release and quantification of carbon disulfide has provided a pragmatic and standardized solution for residue monitoring over the past several decades, this approach is not without its critical limitations. In particular, the lack of chemical specificity: all dithiocarbamates, regardless of their structure, release the same degradation product under acid hydrolysis conditions, thereby having the intrinsically impossibility to identify the original active substance and making it impossible to distinguish between different DTCs, such as mancozeb, thiram, ziram, or propineb, despite their distinct toxicological profiles and environmental behaviors. This analytical ambiguity carries important implications for risk assessment and toxicological evaluation. Each dithiocarbamate possesses unique degradation products, including toxic metabolites such as ethylene thiourea (ETU) and propylene thiourea (PTU), and may exhibit synergistic or additive effects when co-occurring with other residues. By collapsing all DTC residues into a single, non-specific CS_2_ value, current regulatory methods underestimate the complexity of potential health risks and obscure the contribution of individual compounds to cumulative exposure.

In situ detection of CS_2_ has gained attention as a rapid, low-cost alternative to laboratory-based analysis, with techniques such sensor arrays emerging as promising solutions. These methods aim to minimize sample transport and preparation, enabling near-real-time monitoring of dithiocarbamate residues in agricultural fields or food processing environments. Portable electrochemical sensors, photoionization detectors, and fiber-optic probes have been developed for CS_2_ quantification, often with detection limits in the low parts-per-billion range [20]. However, while these in situ platforms offer valuable screening potential, they currently lack the selectivity and reproducibility required for official control programs. Matrix effects, calibration drift, and environmental variability remain major challenges, limiting their utility for legal enforcement or trade dispute resolution. Future integration of in situ methods with confirmatory LC-MS/MS workflows could provide a tiered approach, where field-deployable devices support rapid risk assessment and targeted sampling, while laboratory methods deliver legally defensible results.

In this context, regulatory risk assessment remains inherently conservative, often assuming worst-case scenarios based on arbitrary conversion factors or assumptions of compound identity, which can lead to both over and underestimation of actual toxicological risks. The indirect analytical methods, which involve the determination of CS_2_, are not only unspecific to individual compounds but also poses environmental and safety risks due to the significant amount of organic solvent, mainly iso-octane, and the use of large quantities of concentrated HCl. Furthermore, heating the mixture at high temperatures to facilitate the reaction introduces a safety hazard to manage. In Table 2, the methods involving the hydrolysis of dithiocarbamates and their detection as CS_2_ are reported. In this section, only main information concerning the methods is reported; the detailed information, comprised LOQ and Recovery ranges, for each method is reported in Table S2.

Table 2 provides an overview, with further details in Supplementary Table S1, of all the literature-reported methods involving the determination of dithiocarbamates (DTCs) via their hydrolysis and the subsequent analysis of the carbon disulfide (CS_2_) released during this process. The column titled “Standard Used” refers to the reference compound employed by the authors to monitor the hydrolysis reaction and the corresponding formation of CS_2_. As can be observed, in several cases, such as method 6, no standard was used, resulting in a lack of information regarding the percentage of hydrolysis and, consequently, the precision of the method. In other instances, CS_2_ itself was used as an internal standard, e.g., method 7, which again provides no insight into the completeness of the hydrolysis reaction. In some methods, Thiram was employed as the sole internal standard, e.g., method 1. However, given that Thiram exhibits the highest hydrolysis efficiency among DTCs, as documented in the EURL Single Residue Method (SRM), Version 3.2 (2024) [61], its use does not allow for meaningful conclusions regarding the hydrolysis efficiency of other DTCs. This is particularly relevant for compounds like Ziram, Propineb, and Metiram, which are known to exhibit significantly lower conversion rates and poorer recovery following hydrolysis.

**Figure 4:**
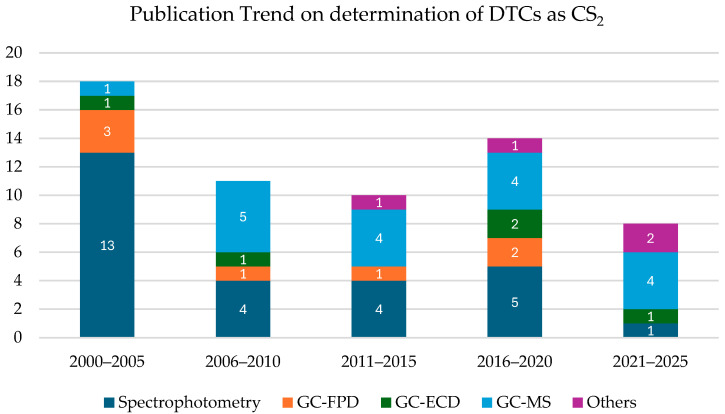
Publication trend from 2000 to 2025 on analytical methods published concerning the determination of dithiocarbamates after their hydrolysis and subsequent formation of CS_2_.

The graph reported in Figure 4 shows the evolution of publications concerning determination of DTCs without involving their hydrolysis and subsequently CS_2_ formation.

The graph illustrates the evolution of publications concerning analytical methods used for the determination of DTCs as CS_2_ from 2000 to 2025. Overall, there has been a gradual decline in the total number of methods published during this 25-year period, indicating a shift away from development of these kinds of methods. Notably, spectrophotometric techniques, which dominated early in the 2000–2005 period with 13 reported methods, have sharply decreased in relevance due to their limited specificity, susceptibility to matrix interferences, and lack of regulatory robustness. In contrast, gas chromatography-based methods, especially GC-MS, have taken the lead in analytical development. Starting modestly in the early 2000s, GC-MS methods have progressively become the preferred approach due to their superior sensitivity, compound confirmation capabilities, and reproducibility. This shift has also been supported by the increased affordability and availability of GC-MS instrumentation over the past two decades, making high-resolution and confirmatory analysis accessible to more laboratories. Furthermore, the global recognition and validation of GC-MS protocols, particularly by regulatory agencies and reference laboratories, has reinforced its position as the gold standard for pesticide residue control and legal enforcement.

### 4.2. Specific Analytical Methods for Single Dithiocarbamates Detection

As awareness of these limitations grew, particularly after 2010, an increasing number of studies have moved beyond total CS_2_ detection toward the selective identification and quantification of individual dithiocarbamates. Researchers began developing and validating compound-specific analytical methods with the aim to bridge the gap between analytical data and toxicological interpretation, providing regulators and public health authorities with more accurate and actionable information. Since the early 2000s, a distinct line of analytical development has emerged focusing on the selective identification and quantification of individual dithiocarbamate residues and their highly toxic metabolites, particularly ethylene thiourea (ETU) and propylene thiourea (PTU), in plant-based foods such as fruits and vegetables [93,122,123].

This approach, fundamentally different from the conventional CS_2_-based methodology, aims to overcome the lack of specificity inherent in indirect detection, offering more accurate assessments of exposure and toxicological risk. The analytical methods developed for single dithiocarbamate determinations mainly involve the use of LC, HPLC, and UPLC coupled with different detectors. The more common configuration provides coupling with MS, especially MS/MS, which provides high sensitivity and structural selectivity needed to distinguish between closely related DTC compounds such as mancozeb, thiram, metiram, ziram, and propineb [2,124]. These MS-based methods have become the gold standard for compound-specific DTC analysis in food, allowing simultaneous detection of parent compounds and their metabolites with LOQs lower than 0.01 mg/kg in several cases, high precision, and satisfying recovery, even in complex plant matrices [125].

Alongside MS detection, other detectors such as spectrophotometers or electrochemical detectors have been occasionally employed, particularly in earlier studies [126]. In addition, some niche methods employed unconventional detectors such as chemiluminescence for detection of Mancozeb and Propineb in cucumbers and apples, with declared LOQ of 0.0003 mg/kg and 0.0014 mg/kg, respectively; or without chromatographic separation, through FAAS detection, with a LOQ of 2 mg/kg for Maneb (detected as Mn) in tomato and wheat grain; and DART or DESI, with recovery rate between 70 and 102% and LOQ > 0.1 mg/kg for Thiram in apples, pears, strawberries, and lettuce [127,128,129].

Emerging biosensors and nanotechnology-based techniques offer exciting opportunities for rapid, on-site pesticide screening, potentially empowering growers, regulators, and consumers with real-time residue data [60]. Nonetheless, these approaches remain primarily research tools; they lack robustness, reproducibility, and regulatory validation required for use in official control laboratories or for legal enforcement. Until their performance is standardized and internationally accepted, they will remain complementary rather than primary analytical tools.

Recently developed methods exploit the reaction of specific dithiocarbamates, e.g., maneb, with Cu^2+^ to form a colored complex that can be quantified by digital image colorimetry following a solid–liquid phase microextraction procedure. Detection is performed by acquiring the intensity of the RGB channels (particularly the blue channel) using a smartphone and a digital analysis application, achieving higher sensitivity compared to conventional spectrophotometric detection. While these approaches are innovative and environmentally friendly, they require further investigation to address potential cross-reactions and interferences that could lead to false positives [130,131].

Most studies on dithiocarbamate (DTC) detection using SERS or other electrode-based sensors are performed under highly controlled laboratory conditions with single-analyte solutions, which do not reflect the complex, multi-residue reality of pesticide contamination reported by EFSA and other surveillance programs. These methods, although undeniably interesting for being environmentally friendly and easy to use, face recurring challenges, including matrix interferences, competitive adsorption, substrate variability, surface fouling, and poor reproducibility, making them unsuitable for legally defensible residue monitoring without confirmatory analysis. In contrast, mass spectrometry-based approaches, although more resource-intensive, provide the selectivity, sensitivity, and robustness needed to characterize DTCs and co-occurring pesticides in real-world samples [46,47].

In Table 3, the main information concerning direct methods developed without involving the hydrolysis of dithiocarbamates is reported. Detailed information for each method, comprised LOQs and Recovery ranges, is reported in Table S2.

Table 3 provides an overview, with further details in Supplementary Table S2, of all the literature-reported methods involving the determination of dithiocarbamates (DTCs) without the evolution of CS_2_. Some of the reported methods for dithiocarbamate determination (e.g., methods 10, 20, 23, 34) involve voltametric techniques or the use of metal nanoparticles. While these approaches can offer high sensitivity, they are generally selective for a specific metal-dithiocarbamate complex and show high specificity. Consequently, their applicability must be individually evaluated for each dithiocarbamate, as performance parameters and interferences may vary depending on the target analyte and matrix [129,136,141,150].

Extraction and stabilization protocols have also evolved to suit the chemical properties of single DTC, often involving alkaline buffers, chelating agents (such as EDTA), and reducing agents to prevent degradation during sample preparation. These method refinements have allowed for more reliable quantification and broader compound coverage compared to the traditional CS_2_-based hydrolysis. These compound-specific approaches not only meet the analytical challenges posed by modern residue monitoring but also provide essential tools for toxicologically meaningful assessments, enabling more nuanced evaluations of dietary exposure and regulatory compliance.

Table 3 finally highlights a critical limitation in the currently available analytical methods for the determination of individual dithiocarbamate analytes. While these methods may demonstrate adequate sensitivity and selectivity under controlled laboratory conditions, they lack sufficient validation data derived from the analysis of real-world samples. This gap restricts the ability to assess the applicability and robustness of the methods in complex matrices typically encountered in food monitoring or environmental surveillance. Furthermore, no data have been reported on the assessment of human exposure to individual pesticides within the dithiocarbamate group based on their respective Maximum Residue Levels (MRLs). This limitation is compounded by the fact that specific MRLs for single fungicides of this chemical class are currently not established in regulatory frameworks, preventing a refined risk assessment for each compound and potentially obscuring compound-specific exposure trends.

**Figure 5:**
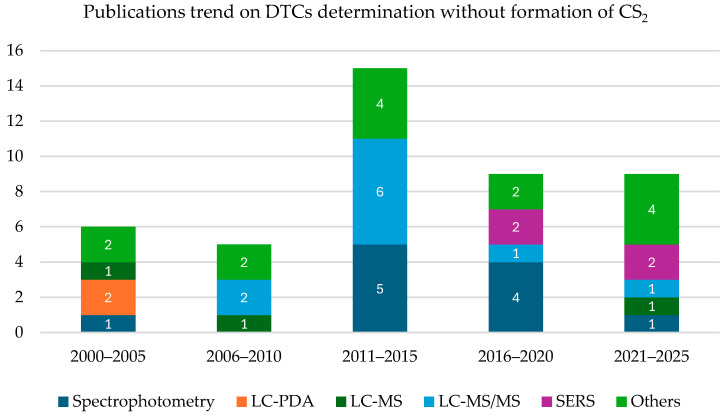
Publication trend from 2000 to 2025 concerning the analytical methods published concerning the determination of dithiocarbamates without the formation of CS_2_.

The graph reported in Figure 5 shows the evolution of publications concerning determination of DTCs without involving their hydrolysis and subsequently CS_2_ formation.

The graph in Figure 5 shows the evolution of publication in the literature concerning analytical methods for DTCs determination without relying on CS_2_ formation from 2000 to 2025. In contrast to methods targeting total CS_2_, publications on compound-specific methods have gradually increased over time, especially peaking between 2011 and 2015. This trend reflects a growing awareness regarding the importance of quantifying individual fungicides rather than relying solely on total CS_2_ measurements, which cannot account for differences in toxicological profiles or for the possible synergistic effects of multiple DTCs. The shift highlights the need for more precise and toxicologically relevant risk assessments. A notable development is the steady rise in LC-MS/MS methods, which now dominate this analytical field due to their unparalleled sensitivity, specificity, and regulatory acceptance. In addition, new experimental methods, such as SERS (surface-enhanced Raman spectroscopy), have gained attention in the last decade for their rapid, on-site analytical potential. However, these emerging technologies remain less versatile and insufficiently validated for legal enforcement, limiting their use to exploratory or research contexts. This reinforces the role of LC-MS/MS as the benchmark technique for high-quality, legally defensible pesticide residue analysis.

## 5. Conclusions and Future Perspectives

The analysis of dithiocarbamate (DTCS) residues in vegetables, fruits, and cereals has long relied on the indirect determination of carbon disulfide (CS_2_) released under acidic hydrolysis. This strategy, established in the 1950s of last century and still upheld in modern regulatory protocols, including EU Regulation 2017/171 and the EURL Single Residue Method (SRM), Version 3.2 (2024), remains the standard reference method across the European Union and many other jurisdictions. However, despite its regulatory acceptance and operational simplicity, the CS_2_-based approach is increasingly recognized as analytically unspecific and toxicologically inadequate. One of the fundamental shortcomings of the CS_2_ method is its inability to discriminate between individual DTC compounds. Since all DTCs degrade into the same analyte, CS_2_, the method inherently overlooks the chemical identity of the original fungicide. This is critical because different DTCs (e.g., Mancozeb, Thiram, Propineb, Ziram, and Metiram) possess distinct toxicological profiles, degradation products, environmental behaviors, and different status regarding authorization in EU. Even more concerning is that the CS_2_ method provides no insight into the levels of highly concerning metabolites such as ethylene thiourea (ETU) and propylene thiourea (PTU), which have been associated with thyroid disruption, potential carcinogenicity, and teratogenicity. Furthermore, the method does not consider any potential synergistic toxicological effects arising from mixtures of DTCs, a situation that is not uncommon in typical agricultural practices. All ethylene-bis-dithiocarbamates (EBDCs) metabolize into ETU, with different rates of production of this metabolite among different DTCs; therefore, exposure scenarios involving combinations such as maneb, mancozeb, and zineb result in a higher cumulative ETU burden than mixtures involving compounds like propineb or thiram. However, CS_2_-based quantification collapses all residues into a single value, ignoring the distinct toxicological contributions of individual fungicides and their potential additive effects. This limitation hampers accurate dietary risk assessments, particularly for populations with high fruit and vegetable consumption, and calls for a paradigm shift towards compound-specific monitoring supported by toxicological modeling.

From an analytical point of view, the current method proposed by EURL SRM v3.2 (December 2024), which uses GC-MS/MS or GC-ECD following cleavage and partitioning into isooctane, has been revised over the years and validated for several matrices, such as plant-origin food, except those with high oil content, and milk. Nonetheless, critical issues remain unresolved. Notably, recovery values for certain compounds such as propineb, metiram, and ziram remain consistently low and highly variable, undermining the reliability and comparability of the results. Moreover, the method requires the use of large volumes of solvents and strong acids, raising environmental and safety concerns. Several efforts to make pesticide residue analysis more sustainable are commendable, with innovations such as sample preparation downscaling, miniaturized extraction protocols, and microextraction techniques, aligning with green chemistry principles and representing meaningful progress toward eco-friendly analytical workflows. However, these methods remain in development, with limited validation across diverse matrices, preventing their integration into standardized regulatory protocols. Continued refinement and harmonization are essential before they can replace solvent-intensive methods currently in use.

Given these shortcomings, there is a growing interest in the scientific and regulatory communities in new developments in specific compound analysis. Over the last two decades, significant progress has been made in developing LC-based methods (HPLC and UPLC) coupled with MS and MS/MS detection, capable of selectively identifying and quantifying individual DTCs and their metabolites in complex food matrices. These direct methods allow for much lower limits of quantification (LOQs), higher analytical accuracy, and most importantly, toxicologically meaningful results. Although such approaches are not yet standardized in legislation, they represent a critical step forward in modernizing residue monitoring. Looking ahead, future analytical strategies should focus on the comprehensive profiling of individual DTCs, including both parent compounds and their known toxic degradation products. This evolution in methodology will not only improve analytical performance but also enable a more accurate assessment of cumulative and synergistic consumer risks. Integrating these compound-specific data into dietary exposure models could support the development of substance-specific MRLs, which reflect both occurrence data and toxicological relevance. Finally, while the CS_2_-based method has served as a practical regulatory tool for decades, its limitations in specificity, toxicological insight, and environmental impact now demand a transition. Embracing direct analytical methods targeting individual DTC residues represents not only a technical advancement but a necessary step toward scientifically robust, health-protective pesticide regulation in the years to come.

The evolution of analytical chemistry for pesticide residue monitoring highlights mass spectrometry as the most promising technique for achieving high specificity and ultra-trace sensitivity in the detection of dithiocarbamates. Unlike SERS or other innovative nanosensor approaches, which remain experimental and lack inter-laboratory validation, mass spectrometry provides a robust, reproducible, and legally defensible framework for regulatory testing. These techniques allow simultaneous multi-residue analysis and accurate quantification of both parent compounds and toxic metabolites such as ETU and PTU, a significant improvement over traditional CS_2_-based methods. Given the legal consequences of pesticide non-compliance and the international trade barriers posed by inconsistent residue reporting, only globally validated, harmonized methods, predominantly based on LC-MS/MS and HRMS, can ensure reliable results across jurisdictions. Thus, while novel techniques are valuable for rapid screening or research, mass spectrometry remains the cornerstone of analytical enforcement.

Chronic dietary exposure to dithiocarbamates represents a particular aspect to be considered for vulnerable populations, especially children, who are more susceptible to endocrine disruptors and neurodevelopmental toxic substances. High consumption of fruits and vegetables, possibly exceeding body weight-adjusted intake in children, enhances this risk. As pesticide use is unavoidable, robust surveillance, stricter enforcement, and proactive risk communication are crucial to safeguard public health, especially among populations with high dietary exposure. For this reason, it is crucial to prioritize the development of innovative analytical methods capable of determining single dithiocarbamate residues with high specificity and sensitivity, moving beyond the current reliance on total CS_2_ quantification. Such methods would enable more accurate exposure assessment, reflecting the distinct toxicological profiles of individual compounds and their metabolites. Equally important is the investigation of potential synergistic or additive effects arising from co-exposure to multiple dithiocarbamates, which are currently underestimated by aggregated CS_2_-based data. A deeper understanding of these interactions will support refined risk assessments and better protect consumers. Ultimately, these advances should inform the establishment of new Maximum Residue Levels (MRLs) for dithiocarbamates based not only on CS_2_ but on compound-specific determinations.

The regulation of dithiocarbamate residues, in fact, remains fragmented globally, with the EU implementing some of the strictest measures, including the phase-out of key fungicides, while Codex Alimentarius and Latin American countries maintain more permissive Maximum Residue Limits (MRLs). The U.S. Environmental Protection Agency (EPA) has adopted compound-specific risk assessments but does not harmonize thresholds across all DTCs, further complicating global trade. This regulatory diversity underscores the urgent need for international standardization of analytical protocols and MRLs to facilitate safe food commerce while ensuring consumer protection.

## Figures and Tables

**Table 1:** Toxicological profile of DTCs and relative main metabolites.

Substance	Main Endpoints	Human Effects	Main Metabolites	Oral LD50 mg/kg (Rat)	References
Ferbam	Hepatotoxicity, Nephrotoxicity, Skin/Eye Irritation	Nausea, Headache, Respiratory Irritation, Chronic Liver and Thyroid Alterations	CS_2_, ETU	~3300	[42,45,49]
Mancozeb	Thyroid Alterations, Reproductive/Developmental Toxicity, Neurotoxicity	Acute Irritation, Subclinical Hypothyroidism, Neurobehavioral Changes	CS_2_, ETU	8000–10,000	[42,45,52]
Maneb	Thyroid Alterations, Neurotoxicity (Experimental Parkinsonism)	Tremors, Cognitive and Thyroid Disturbances in Chronic Exposure	CS_2_, ETU	5000–8000	[42,45,53,54]
Metiram	Thyroid Toxicity, Reproductive Toxicity (Through ETU Formation)	Endocrine and Developmental Effects	CS_2_, ETU	>6000	[42,43,45]
Propineb	Skeletal Abnormalities, Thyrostatic Effect, Hepatotoxicity (Through PTU Formation)	No Direct Human Clinical Studies	CS_2_, PTU	>5000	[42,50,51]
Thiram	Potential Neurotoxicity, Hepatoxicity, Dermal Irritation, Nephrotoxicity	Drowsiness, Confusion, Loss of Sex Drive, Incoordination, Slurred Speech, Weakness, Dermatitis, Conjunctivitis, Nausea, Cardiovascular Disturbances	CS_2_	210–2000	[38,39,41,42]
Zineb	Thyroid Toxicity, Reproductive Toxicity (Through ETU Formation)	Tiredness, Dizziness, Weakness, Headache, Nausea, Fatigue	CS_2_, ETU	1850–8900	[42,44,45]
Ziram	Potential Neurotoxicity, Dopaminergic Neuron Toxicity	Reproductive Effects	CS_2_	400–480	[46,47,48]
ETU (Ethylene thiourea)	Thyroid toxicity, Goitrogenic, Experimental Carcinogenicity, Reproductive Toxicity	Hypothyroidism, Thyroid Hormone Disruption	—	1800–2000	[42,45]
PTU (Propylene thiourea/Propylthiouracil)	Thyrostatic Effect, Hepatotoxicity, Potential Mutagenicity	Hypothyroidism, Goiter, Hepatotoxicity	—	1300–1500	[42,57]
CS_2_ (Carbon disulfide)	Neurotoxicity (CNS and PNS), Cardiovascular Effects, Reproductive Toxicity	Peripheral Neuropathy, Cognitive/Mood Disorders, Accelerated Atherosclerosis, Acute Headache/Dizziness	Thiocarbonates, Protein-bound Compounds	1200–3000	[42,58]

**Table 2:** Methods reported in the literature from 2000 to 2025, involving the formation of CS_2_ for dithiocarbamate quantitative determination (for details on methods see Table S1).

Method	Standard Used	Matrix	Chromatographic Method	Detection Method	Year	Author [Ref]
1	Thiram	Tree Nuts, Celeriac, Endive, Carrot, Radish, Onion, Garlic, Shallot, Cucumber, Cherries, Plums, Cauliflower, Apple, Lettuce	None	UV (435 nm)	2000	Heise et al. [70]
2	Carbon Disulfide	Savoy Cabbage, Red Cabbage, Turnip-Rooted Cabbage, Cauliflower, Leek, Table Mustard	None	UV-Vis (240–360 nm)	2000	Perz et al. [17]
3	Zineb	Apple, Apricot, Blueberry, Cherry, Grape, Nectarine, Peach, Pear, Plum, Raspberry, Rhubarb, Strawberry, Asparagus, Beans, Broccoli, Cabbage, Carrot, Cauliflower, Celery, Cucumber, Lettuce, Mushroom, Onion, Parsnip, Pepper, Potato, Radish, Tomato, Specialty	None	UV (435 nm)	2000	Ripley et al. [84]
4	Thiram	Apple, Papaya, Orange, Banana, Dry Beans, Polished Rice, Potato, Tomato, Cucumber	None	UV (435 nm)	2001	Caldas et al. [69]
	Mancozeb					
	Ziram					
5	Sodium Diethyldithiocarbamate	Grape leaf, Lettuce, Cantaloupe, Cucumber, Eggplant, Green Beans, Green Peas, Pepper, Tomato, Apple, Grape, Peach, Strawberry	None	UV (435 nm)	2001	Dogheim et al. [71]
6	None	Apple, Grape, Orange, Tomato, Eggplant, Cucumber, Potato	None	UV (435 nm)	2001	Abbassy et al. [72]
7	Carbon Disulfide	Apple, Apricot, Aubergine, Beans, Carrot, Cherry, Cucumber, Black Currant, Red Currant, Dill, Gooseberry, Grape, Kumquat, Leek, Lettuce, Melon, Nectarine, Okra, Oregano, Papaya, Parsley, Passion Fruit, Peas, Peach, Pear, Pepper, Plum, Raspberry, Spinach, Onion, Strawberry, Tomato, Watermelon	None	UV (372, 430 nm)	2001	Andersen et al. [73]
8	Sodium Diethyldithiocarbamate	Cucumber, Green Peas, Pepper, Tomato, Grape, Peach, Strawberry	None	UV (435 nm)	2002	Dogheim et al. [85]
9	Carbon Disulfide	Cucumber, Lettuce, Tomato, Mushroom, Red beet, Sugar beet, Cherry, Currant, Plum, Raspberry, Strawberry,	None	UV (662 nm)	2002	Morzycka et al. [86]
10	Zineb	Tobacco (T), Peach (P)	GC	FPD	2002	Vryzas et al. [87]
	Mancozeb					
	Ziram					
	Maneb					
	Thiram					
11	Carbon Disulfide	Grape Leaf, Lettuce, Molokhia, Spinach, Cantaloupe, Cucumber, Eggplant, Green Beans, Green Peas, Pepper, Tomato, Apple, Grape, Lemon, Lime, Peach, Pear, Strawberry	None	UV (435 nm)	2002	Gad Alla et al. [88]
12	Carbon Disulfide	Apple, Apricot, Asparagus, Avocado, Banana, Basil, Beans, Beetroot, Blackberry, Broccoli, Carambola, Carrot, Celery, Cherry, Chili, Cucumber, Black Currant, Red Currant, Dandelion leaves, Fennel, Fig, Grapefruit, Kaki, Kale, Kiwi, Kumquat, Lemon, Lettuce, Mandarin, Mango, Melon, Orange, Papaya, Passion Fruit, Peach, Pear, Pepper, Pineapple, Pomegranate, Potato, Radish, Rambutan,, Raspberry, Spinach, Onion, Strawberry, Tomato, Watercress	None	UV (372, 430 nm)	2003	Poulsen et al. [74]
13	None	Tomato, Plum, Strawberry, Currant, Lettuce, Mushrooms, Cherry, Cucumber, Apple	None	UV (662 nm)	2003	Nowacka A. [89]
14	Mancozeb	Apple, Banana, Orange, Papaya, Strawberry, Potato, Tomato, Dry Beans, Rice	None	UV (435 nm)	2004	Caldas et al. [90]
	Ziram					
	Thiram					
15	Ziram	Courgette	GC	MS	2004	Guidotti M. et al. [91]
16	Carbon Disulfide	Raspberry	GC	ECD	2004	Kovacevic et al. [92]
17	Maneb	Tomato	GC	FPD	2004	Kontou et al. [93]
18	Carbon Disulfide	Chinese Chive, Carrot, Spinach, Lettuce, Grape, Chinese Mustard, Celery, Radish, Ching Geeng, Wax apples, Cabbage, Bitter melon, Green Pepper	GC	FPD	2005	Chang et al. [94]
19	N.R.	Apple, Lettuce, Potato	GC	MS	2006	Česnik et al. [95]
20	None	Apple, Tomato, Papaya, Lettuce, Strawberry, Banana, Orange, Carrot, Potato, Beans, Rice	None	UV (435 nm)	2006	Caldas et al. [76]
21	Thiram	Apple, Lettuce, Potato, Strawberry, Tomato	GC	MS	2006	Česnik et al. [96]
22	Thiram	Peach, Green Beans, Apple, Tomato, Green Pepper, Potato, Fruit Cream Powder, Dehydrated vegetable cub	GC	FPD	2006	Papadopoulou-Mourkidou et al. [97]
	Mancozeb					
23	None	Apples, Cauliflower, Cereals, Grapes, Lettuce, Peas, Peeper, Potato, Strawberries	GC	MS	2007	Česnik et al. [98]
24	Ziram	Apple, Pear, Cherry, Grape, Tomato, Cucumber, Tamarillos, Papaya, Broccoli	GC	MS	2008	Crnogorac et al. [63]
	Carbon Disulfide					
	Dithiane					
	Antracol					
25	Mancozeb	Green Beans	None	UV (435 nm)	2009	Bazzi et al. [99]
	Mefenoxam					
26	None	Fig, Mango, Papaya, Persimmon	None	UV (435 nm)	2009	Pastor Ciscato et al. [77]
27	None	Tomato, Cabbage, Lettuce, Cucumber, Carrot, Spinach, Potato, Onion, Pepper	GC	ECD	2009	Lazic et al. [100]
28	Thiram	Tangerine, Clementine, Orange, Peach, Nectarine, Khakis	GC	MS	2009	Berrada et al. [101]
	Zineb					
	Ziram					
29	Maneb	Grape, Strawberry, Carrot, Lettuce, Corn	VP-LPME	IR	2010	Gonzalvez et al. [82]
	Ziram		GC	MS		
	Mancozeb					
30	Carbon Disulfide	Grape Leaf, Lettuce, Cantaloupe, Cucumber, Eggplant, Green Beans, Green Peas, Pepper, Tomato, Apple, Grape, Peach, Strawberry, Squash, Broccoli, Potato, Apricot, Orange, Plum, Watermelon	None	UV (435 nm)	2010	Khorshed et al. [102]
	Sodium Diethyldithiocarbamate					
31	Carbon Disulfide	Green Beans, Green Peas, Broccoli, Green Onion, Potato Leaves, Peanut	GC	MS	2011	El-Gohary et al. [103]
	Sodium Diethyldithiocarbamate					
32	None	Apple	None	UV (435 nm)	2011	Łozowicka et al. [104]
33	Thiram	Apple, Leek, Potato, Strawberry, Wheat, Tomato, Lettuce, Rice	GC	FPD	2012	Bempelou et al. [105]
34	Carbon Disulfide	Watermelon, Banana, Mango, Cauliflower, Potato, Apricot, Grape, Green Peas, Lettuce, Molokhia, Watercress, Cucumber, Eggplant, Squash, Tomato, Cantaloupe, Guava, Strawberry, Spinach, Grape Leaf	None	UV (435 nm)	2012	El-Sawi Sanaa A. et al. [106]
	Sodium Diethyldithiocarbamate					
35	Mancozeb	Raspberry	GC	MS	2012	Pucarevic et al. [107]
36	Carbon Disulfide	Tomato, Paprika, Cucumber, Potato, Onion, Carrot, Cabbage, Ketchup, Apple, Cherry, Grape, Wine	GC	MS	2014	Kostik et al. [108]
37	Mancozeb	Grape, Green Chili, Tomato, Potato, Brinjal, Pineapple, Chayote	GC	MS	2014	Mujawar et al. [109]
38	None	Apple, Blueberry, Currant, Raspberry, Tomato, Broccoli, Parsley, Cucumber, Cabbage	None	UV (435 nm)	2015	Szpykra E. et al. [110]
39	None	Apple, Black Berries, Chokeberries, Blueberries, Currants, Elderberries, Gooseberries, Pears, Plums, Raspberries, Sea Sallowthorns, Cherries, Strawberries	None	UV (662 nm)	2015	Łozowicka et al. [111]
40	None	Apple, Apricot, Black Currant, Gooseberry, Grape, Peach, Raspberry, Red Currant, Strawberry, Sweet Cherry, Broccoli, Brussels Sprout, Carrot, Celeriac, Dill, Lettuce, Parsley, Peaking Cabbage, Spinach, Tomato, Wheat	None	UV (435 nm)	2016	Szpykra E. et al. [112]
41	Mancozeb	Tomato	None	UV (435 nm)	2017	El Habib Ait Addi et al. [113]
42	Mancozeb	Tomato	GC	MS	2017	Atuhaire et al. [114]
43	Mancozeb	Leafy Vegetables (Lettuce, Chard, Spinach)	None	UV-Vis (240–360 nm)	2017	Elgueta et al. [75]
44	Thiram	Lettuce	GC	ECD	2017	Pizzutti et al. [115]
			GC	PFPD		
			GC	MS		
			None	UV (435 nm)		
45	Mancozeb	Rice, Corn, Cabbage	GC	MS	2017	Shao et al. [79]
46	Propineb and metabolites (PDA-PTU)	Banana	GC	FPD	2017	Song et al. [116]
	Carbon Disulfide					
47	Carbon Disulfide	Apricot	GC	MS	2018	Arslan et al. [117]
48	Zineb	Eggplant, Broccoli, Potato, Pear, Onion, Cabbage, Lettuce, Spinach, Lettuce, Ginger, Pepper, Cucumber, Cowpea, Tomato, Orange, Pumpkin, Strawberry, Banana, Papaya, Guava, Star Fruit, Watermelon, Apple, Radish, Carrot	LC	DBD-MES	2018	Han et al. [83]
49	Carbon Disulfide	Tomato, Mango, Cabbage, Grape	GC	ECD	2018	Nguyen et al. [80]
50	Thiram	Passion Fruit	None	UV (435 nm)	2019	Mozzaquatro et al. [118]
51	Mancozeb	Onion, Onion Leaves	GC	MS	2019	Patil et al. [119]
52	Carbon Disulfide	Apple, Avocado, Papaya, Durian, Soursop, Lemon, Guava, Mango, Orange, Mangosteen, Passion Fruit, Pineapple, Pomelo, Banana, Pitaya, Rambutan, Rockmelon, Salacca, Watermelon, Jackfruit	GC	FPD	2019	Rahman Alinah A. [120]
53	Mancozeb	Lettuce	None	Vis-NIR (600 µm)	2020	Steidle Neto et al. [20]
54	Thiram	Soybean	GC	ITD-MS	2021	da Silva et al. [21]
				PFPD		
55	None	Arugula, Bean pod, Bean root, Carrot, Chayote, Chicory, Chili, Coriander, Chive, Eggplant, Ginger Leek, Lettuce, Parsley, Pumpkin, Scarlet eggplant, Spinach, Sweet pepper, Sweet potato, Tomato, Watercress, Yam, Zucchini	None	UV (435 nm)	2022	de Araujo et al. [121]
56	Carbon Disulfide	Vine Leaves	GC	MS	2022	Arslan et al. [22]
57	Carbon Disulfide	Cardamom, Black Pepper	GC	MS	2022	Natarajan et al. [23]
58	Thiram	Yerba Mate	GC	MS	2022	Da Silva [24]
59	Mancozeb	Mango, Banana, Rice, Cowpea, Lychee, Cabbage	LC	LEGD-DBD-MES (257.94 nm)	2023	Tian et al. [26]
	Metiram					
	Thiram					
	Propineb					
60	Mancozeb	Banana, Mango, Pineapple, Cowpea, Dragon Fruit, Lychee, Apple, Eggplant, Peanuts	GC	ECD	2024	Tian et al. [25]
61	Mancozeb	Cauliflower	HPLC	MS-MS	2025	Tripathi et al. [27]

**Table 3:** Methods reported in the literature from 2000 to 2025, without formation of CS_2_ for dithiocarbamate quantitative determination (for details on methods see Table S2).

Method	Target Compounds	Matrix	Chromatographic Method	Detection Method	Year	Author [Ref]
1	Ziram	Potato, Cabbage, Tomato, Cucumber	None	UV (590 nm)	2001	Saad et al. [126]
2	Ethylene thiourea	Tomato, Tomato Juice, Tomato Paste	HPLC	PDA	2001	Kontou et al. [122]
3	Dazomet	Avocado, Cherry, Lemon, Nuts, Oat, Orange, Peach, Rice, Tomato	LC	APCI-MS	2003	Blasco et al. [132]
	Disulfiram					
	Thiram					
	Ethylene thiourea					
	Propylene thiourea					
4	Ethylene thiourea	Tomato	HPLC	PDA	2004	Kontou et al. [93]
5	Mancozeb	Cucumber, Apple	HPLC	CL	2004	Nakazawa et al. [127]
	Propineb					
6	Maneb	Tomato, Wheat Grain, Water	None	FAAS	2005	Turker et al. [128]
7	Ziram	Grapes, Cucumbers, Tomatoes, Rucola	HPLC	MS	2007	Crnogorac et al. [133]
	Dithiane					
	Antracol					
	Zineb					
	Propineb					
8	Ziram	Apple, Pear, Cherry, Grape, Tomato, Cucumber, Tamarillos, Papaya, Broccoli	HPLC	MS-MS	2008	Crnogorac et al. [63]
	Dithiane					
	Antracol					
9	Mancozeb	Persimmons, Pears, Strawberries, Cabbage, Lettuce, Spinach	HPLC	MS-MS	2008	Hayama et al. [134]
	Maneb					
	Zineb					
10	Ziram	Potato, Cabbage, Tomato	None	Square Wave Voltammetry (SWV)	2008	Qiu et al. [135]
11	Ziram	Cacao, Spinach, Potato, Brown Rice, Pumpkin, Orange, Soybean, Cabbage, Apple, Green Tea	GC	MS	2010	Nakamura et al. [136]
	Thiram					
	Ferbam					
	Nickel bis(dithiocarbamate)					
	Propineb					
	Maneb					
	Zineb					
	Mancozeb					
	Polycarbamate					
	Milneb					
12	Ethylene thiourea	Celery, Melon, Spinach	UHPLC	MS-MS	2011	Bonnechere et al. [123]
	Propylene thiourea					
13	Thiram	Apple, Pear, Strawberry, Lettuce	DART	MS	2011	Cajka et al. [129]
	Ziram		DESI	MS-MS		
14	Thiram	Tomato	HPLC	UV (272 nm)	2012	Jafari et al. [137]
	Mancozeb					
	Propineb					
15	Thiram	Eggplant, Lettuce, Strawberry, Apple	HPLC	MS-MS	2012	Peruga et al. [124]
16	Ethylene thiourea	Rice, Leaf Mustard	UPLC	MS-MS	2012	Chung et al. [138]
	Propylene thiourea					
17	Mancozeb	Apple, Wine Grape, Lettuce, Pepper, Tomato, Strawberry	HPLC	UV (270 nm)	2012	Lopez-Fernandez et al. [139]
	Maneb					
	Propineb					
18	Antracol	N.A.	MALDI	MS (Orbitrap)	2013	Ivanova et al. [140]
	Ferbam					
	Maneb					
	Mancozeb					
	Propineb					
	Thiram					
	Ziram					
19	Thiram	Tomato, Grape, Sweet Peppers, Nectarine, Peach	HPLC	MS-MS	2013	Ringli et al. [141]
20	Mancozeb	N.A.	None	Adsorptive Stripping Voltammetry (AdSV)	2013	Amorello et al. [142]
	Maneb					
	Propineb					
	Nabam					
	Na (CH3)2DTC					
	Zineb					
	Ziram					
	Ferbam					
	Thiram					
21	Thiram	Tomato, Cucumber, Watermelon	None	UV (430 nm)	2013	Rastegarzadeh et al. [143]
22	Ferbam	Apple, Pear, Plum, Grape, Papaya, Broccoli, Tomato	HPLC	MS-MS	2013	Schmidt et al. [144]
	Mancozeb					
	Maneb					
	Metiram					
	Nabam					
	Propineb					
	Thiram					
	Zineb					
	Ziram					
23	Mancozeb	Fruit Juice, Water	None	UV (620 nm)	2014	Rohit et al. [145]
				HNMR		
				FT-IR		
				TEM		
24	Ethylene thiourea	Apple, Papaya, Strawberry	HPLC	MS-MS	2014	Rossi Lemes et al. [146]
25	Disulfiram	Apple, Grape, Lettuce	UHPLC	ED	2015	Charoenkitamorn et al. [147]
	Thiram					
	N,N-diethyl-N′,N′-dimethyl thiuram disulfide					
26	Dazomet	Apple, Leek, Tomato, Pine needles	HPLC	UV (272 nm) AAS	2016	Al-Alam et al. [148]
	Metam Sodium					
	Ferbam					
	Ziram					
	Zineb					
	Maneb					
	Mancozeb					
	Metiram					
	Nabam					
	Propineb					
27	Propineb	Beer, Fruit Juice, Malt	HPLC	MS-MS	2017	Kakitani et al. [149]
	Mancozeb					
	Maneb					
	Zineb					
	Polycarbamate as EB					
	Milneb					
	Thiuram					
	Nickel diethyldithiocarbamate					
	Polycarbamate as DD					
	Ferbam					
	Ziram					
28	Propineb	Banana	GC	FPD	2017	Song et al. [116]
	Propylene thiourea		HPLC	MS-MS		
	Propylene diamine					
29	Thiram	Strawberry, Cucumber	None	SERS	2018	Chen et al. [150]
30	Ziram	Water, Tomato, Mango Beverage	None	UV-Vis (400–570 nm)	2019	Ghoto et al. [151]
	Zineb					
	Maneb					
31	Ziram	Water, Tomato, Mango Beverage	None	UV-Vis (490–570 nm)	2019	Ghoto et al. [152]
	Zineb					
	Maneb					
32	Ziram	Apple Black Tea	None	UV-Vis (525–683 nm)	2020	Wang et al. [28]
	Thiram					
	Zineb					
33	Propineb	Infant Formula, Black tea	GC	MS	2020	Bodur et al. [153]
34	Ziram	Apple Juice	None	SERS	2020	Wei et al. [29]
	Thiram					
35	Thiram	Lettuce, Broccoli	None	SERS	2021	Tsen et al. [30]
	Mancozeb					
	Propineb					
36	Thiram	Tap Water, Orange Juice	None	SERS	2022	Ahn et al. [31]
37	Mancozeb	Chamomile	HPLC	MS-MS	2022	Sayed et al. [34]
38	Ethylene thiourea	Grape, Cherry Tomato, Strawberry	HPLC	ICP-MS	2023	Bendhiab et al. [35]
	Propylene thiourea					
39	Thiram	Water, Apple, Guava, Broad Beans, Green Beans	None	UV-Vis (420 nm)	2023	Eswaran et al. [154]
40	Ethylene thiourea	Cucumber, Celery, Tomato, Green pepper, Potato, Citrus, Apple, Jujube, Raisins, Grape	None	Fluorescence (450–570 nm)	2023	Han et al. [32]
41	Maneb	Tomato, Rice, Papaya	None	Digital Images	2023	Martins et al. [131]
42	Ziram	Green tea, Flower tea, Red tea, Black tea	HPLC	CDCL	2024	Wei et al. [155]
	Zineb					
	Propineb					
43	Zineb	Green Tea, White Tea, Black Tea	None	Fluorescence (500–650 nm)	2025	Feng et al. [33]
				UV (365 nm)		

N.A.—Not Applicable.

## Data Availability

The data presented in this study are available on request from the corresponding author.

## Supplementary Materials

The following supporting information can be downloaded at https://www.mdpi.com/article/10.3390/toxics13100819/s1; Table S1: Analytical methods published in years 2000–2025 for determination of dithiocarbamates after hydrolysis and CS_2_ formation, detailed information; Table S2: Analytical methods published in years 2000–2025 for determination of dithiocarbamates without CS_2_ formation, detailed information.
