# Supplementary material for: Advances in the Analytical Determination and Toxicological Assessment of Dithiocarbamates and Their Hydrolysis Products in Fruits, Vegetables, and Cereals: Methodological Evolution, Challenges, and Future Directions
Source: Toxics. 2025 Sep 26;13(10):819. doi: 10.3390/toxics13100819 (PMC12567660; doi:10.3390/toxics13100819)
Supplement: Supplementary file 1 [file toxics-13-00819-s001.zip › toxics-3847324-supplementary.pdf]

Table S1. Analytical methods published in years 2000-2025 for determination of dithiocarbamates after hydrolysis and CS_2 formation, detailed information.

#	Standard Used	Matrix	Reagents	Chromatographic Method	Detection Method	Samples Analyzed	Recovery Ranges	Ranges found (mg/kg)	Samples Above LOQ	Samples Above MRL	MLR (dithiocarbamates expressed as CS_2, mg/Kg)	Location	Year	Author	Leading Institute	Reference (DOI, PMID, ISBN, CABI, URL, Others)	Notes	Reference Regulation
**1**	Thiram	Tree Nuts, Celeriac, Endive, Carrot, Radish, Onion, Garlic, Shallot, Cucumber, Cherries, Plums, Cauliflower, Apple, Lettuce	$SnCl_2$; HCl; NaOH; $Pb(CH_3COO)_2$; $Cu(CH_3COO)_2$; Diethanolamine; Methanol	None	UV (435 nm)	N.A.	80-100%	N.A.	N.A.	N.A.	0.1 (Tree Nuts) 0.2 (Carrots, Celeriac) 0.5 (Garlic, Shallot, Onion, Cucumber) 3 (Apples) 1 (Plums, Cherries, Cauliflower) 5 (Lettuce, Radish, Endive)	Germany	2000	Heise et al.	Federal Institute for Consumer Health Protection and Veterinary Medicine, Berlin	DOI: 10.1007/s002160051584	Cullen and Keppel Method	Directive 98-82EC 1998
**2**	Carbon Disulfide	Savoy Cabbage, Red Cabbage, Turnip-Rooted Cabbage, Cauliflower, Leek, Table Mustard	HCl; $SnCl_2$; KOH; Methanol, $Zn(CH_3COO)_2$; Ethylenediamine	None	UV (240-360 nm)	83	N.A.	2.8-3.3	72 (> 0.05 mg/kg)	12	1.0 (Cabbage vegetables)	Germany	2000	Perz et al.	Institute of Food Chemistry, University of Hohenheim	DOI: 10.1021/jf9905323	Xanthate Method	Directive 98-82EC 1998
**3**	Zineb	Apple, Apricot, Blueberry, Cherry, Grape, Nectarine, Peach, Pear, Plum, Raspberry, Rhubarb, Strawberry, Asparagus, Beans, Broccoli, Cabbage, Carrot, Cauliflower, Celery, Cucumber, Lettuce, Mushroom, Onion,	NaOH, Benzene, $SnCL_2$, HCl, Ethanol, Diethanolamine, Toluene, Na_2SO_4, NaOH	None	UV-Vis (435 nm)	2338	70-110%	0.06-35	755 (>0.05 mg/kg)	41	EBDC, Ferbam and Ziram respectively: 0.1,0.1,0.1 (Parsnip, Potato) 7, 0.1,0.1 (Mushroom) 0.1, 7, 7 (Apricot, Cherry, Peach, Plum, Raspberry,	Canada	2000	Ripley et al.	University of Guelph, Laboratory Services Division, Pesticide and Trace Contaminants Laboratory, Guelph	PMID: 10693021	Cullen Method	Health and Welfare Canada (1991 rev.) Food and Drugs Act and Regulations, Supply and Services Canada, Hull, Quebec, Canada

		Parsnip, Pepper, Potato, Radish, Tomato, Specialty									Strawberry, Asparagus, Beans, Beets, Carrot, Radish Spinach)<br>4, 7, 7 (Tomato)<br>5,7,7 (Celery)<br>7, 7, 7 (Apple, Grape, Pear, Broccoli, Cabbage, Cauliflower, Lettuce, Onion, Pepper)							
**4**	Thiram<br>Mancozeb<br>Ziram	Apple, Papaya, Orange, Banana, Dry Beans, Polished Rice, Potato, Tomato, Cucumber	$SnCl_2$; HCl; NaOH; $Pb(CH_3COO)_2$; $Cu(CH_3COO)_2$; Diethanolamine; Methanol	None	UV-Vis (435 nm)	100	82-120%	0.10-6	N.A.	N. A.	0.1 (Apple, Potato, Banana)<br>0.5 (Cucumber)<br>2 (Tomato)	Brazil	2001	Caldas et al.	Department of Pharmaceutical Sciences, College of Health Sciences and Chemistry Institute, University of Brasilia	DOI: 10.1021/jf010124a	Caldas Method (Improvement to Cullen and Keppel Methods)	ANVISA, Agencia Nacional de Vigilancia Sanitaria (Br); Codex alimentarius (1994)
**5**	Sodium Diethyl DTC	Grape leaf, Lettuce, Cantaloupe, Cucumber, Eggplant, Green Beans, Green Peas, Pepper, Tomato, Apple, Grape, Peach, Strawberry	$SnCl_2$; HCl; NaOH; $Pb(CH_3COO)_2$; $Cu(CH_3COO)_2$; Diethanolamine; Ethanol	None	UV-Vis (435 nm)	510	80-110%	0.1-10	48 (>0.2 mg/kg)	2	0.5 (Cucumber, Green Beans)<br>1 (Grape, Grape leaf, Cantaloupe, Eggplant, Green Peas, Pepper, Peach)<br>3 (Tomato, Apple, Pear, Strawberry)<br>5 (Lettuce)	Egypt	2001	Dogheim et al.	Ministry of Agriculture, Central Laboratory of Residue Analysis of Pesticide and Heavy Metals in Food, Dokki	DOI: 10.1093/jaoac/84.2.519	Keppel Method	MRL by Codex Committee of Pesticides Residues unless indicated as EU (European MRLs)
**6**	None	Apple, Grape, Orange, Tomato, Eggplant, Cucumber, Potato	HCl; $SnCl_2$; $Cu(CH_3COO)_2$, Diethanola	None	UV-Vis (435 nm)	78	76-95%	0.02-0.87	72 (>0.02 mg/kg)	0	N.A.	Egypt	2001	Abbassy et al.	Department of Environmental Studies, Institute of	DOI: 10.1007/s00128-001-0114-y	Cullen and Keppel Method	CODEX Alimentarius Commission 1991.

			mine, Ethanol, NaOH												Graduate Studies and Research, University of Alexandria			
**7**	Carbon Disulfide	Apple, Apricot, Aubergine, Beans, Carrot, Cherry, Cucumber, Black Currant, Red Currant, Dill, Gooseberry, Grape, Kumquat, Leek, Lettuce, Melon, Nectarine, Okra, Oregano, Papaya, Parsley, Passion Fruit, Peas, Peach, Pear, Pepper, Plum, Raspberry, Spinach, Onion, Strawberry, Tomato, Watermelon	$SnCl_2$, HCl, $Cu(CH_3COO)_2$, Diethanolamine	None	UV-Vis (372, 430 nm)	4150	70-110%	0.1-22	148 (>0.1 mg/kg)	38	0.05 (Kumquat, Papaya, Passion Fruit, Spinach) 0.3 (Carrot) 0.5 (Cucumber, Onion) 1 (Beans, Cherry, Melon, Okra, Peas, Plum, Watermelon) 2 (Apple, Apricot, Aubergine, Currant, Gooseberry, Grape, Leek, Nectarine, Peach, Pear, Pepper, Raspberry, Strawberry) 3 (Tomato) 5 (Dill, Lettuce, Oregano, Parsley)	Denmark	2001	Andersen et al.	Danish Veterinary and Food Administration, Soborg	DOI: 10.1080/02652030110054759	Juhler Method (1999)	European Union, 1997a, Commission Directive 97/71/EC of 15 December 1997 amending the Annexes to Council Directives 86/362/EEC, 86/363/EEC and 90/642/EEC on the fixing of maximum levels for pesticide residues in and on cereals, foodstuffs of animal origin and certain products of plant origin, including fruit and vegetables respectively.
**8**	Sodium Diethyl DTC	Cucumber, Green Peas, Pepper, Tomato, Grape, Peach, Strawberry	$SnCl_2$; HCl; NaOH; $Pb(CH_3COO)_2$; $Cu(CH_3COO)_2$; Diethanolamine; Ethanol	None	UV-Vis (435 nm)	318	80-110%	0.1-10	21 (>0.2 mg/kg)	1	0.5 (Cucumber) 1 (Pepper) 3 (Peach, Strawberry, Tomato) 5 (Grape)	Egypt	2002	Dogheim et al.	Ministry of Agriculture, Central Laboratory of Residue Analysis of Pesticide and Heavy Metals in Food, Dokki	DOI: 10.1080/02652030210157655	Keppel Method	MRL by Codex Committee of Pesticides Residues unless indicated as EU (European MRLs)

**9**	Carbon Disulfide	Cucumber, Lettuce, Tomato, Mushroom, Red beet, Sugar beet, Cherry, Currant, Plum, Raspberry, Strawberry,	HCl; $SnCl_2$; KOH; Methanol, $Zn(CH_3COO)_2$; p-Dimehtylamino aniline; Iron (III)	None	Vis (662 nm)	2226	78-98%	0.05-27.5	252 (>0.05 mg/kg for Mushrooms, >0.2 mg/kg for Lettuce, >0.1 mg/kg for others)	20	0.05 (Mushrooms) 0.5 (Cucumber) 1 (Cherry, Plum) 2 (Strawberry) 3 (Tomato) 5 (Lettuce, Currant)	Poland	2002	Morzycka et al.	Institute of Plant Protection, Regional Experimental Station Białystok	YADDA: bwmeta1.element.agro-article-5894b695-85a7-4574-b0ac-30053407217d	Xanthate Method: 6 more tomato samples and 2 more currant samples are above MRLs for Poland regulation, which are in these cases lower than EU limits	MRL by Council Directives 86/362/EEC and 90/642/EEC with amendments up to 2001/57/EC
**10**	Zineb<br>Mancozeb<br>Ziram<br>Maneb<br>Thiram	Tobacco (T), Peach (P)	HCL, $SnCl_2$, Isooctane, EDTA, Acetone, Pyridine, MW	GC	FPD	>3000 (T) N.R. (P)	81-142%	0.2-45 (T) 0.01-0.1 (P)	N.A. (>0.006 mg/kg for peaches; >0.2 mg/kg for tobacco)	N.A.	N.A. (Tobacco) 3 (Peach)	Greece	2002	Vryzas et al.	Pesticide Science Laboratory, Aristotle University, Thessaloniki	DOI: 10.1021/jf0111864	Assisted MW method, Validation, no info concerning the number of peach samples were reported in the document	No MRL provided at that time for Tobacco; for peaches: MRL by Codex Committee of Pesticides Residues unless indicated as EU (European MRLs)
**11**	Carbon Disulfide	Grape Leaf, Lettuce, Molokhia, Spinach, Cantaloupe, Cucumber, Eggplant, Green Beans, Green Peas, Pepper, Tomato, Apple, Grape, Lemon, Lime, Peach, Pear, Strawberry	Ethanol, Diethanolamine, HCl, Toluene, Na_2SO_4, NaOH, $SnCl_2 \cdot 2H_2O$, $Cu(CH_3OOH)_2$	None	UV-Vis (435 nm)	1414	80-110%	0.1-6.8	154 (>0.05 mg/kg)	4	0.5 (Cucumber, Green Beans, Green Peas) 1 (Cantaloupe, Pepper) 2 (Lemon, Lime) 3 (Eggplant, Apple, Peach, Peas, Strawberry, Tomato) 5 (Grape, Lettuce)	Egypt	2002	Gad Alla et al.	Central Laboratory of Residue Analysis of Pesticides and Heavy Metals in Food, Agricultural Research Center, Dokki, Giza	ISSN: 1110-6336 CABI Record Number: 20033073770	Keppel Method	CCPR Codex Committee of Pesticide Residues 1993, 1999

12	Carbon Disulfide	Apple, Apricot, Asparagus, Avocado, Banana, Basil, Beans, Beetroot, Blackberry, Broccoli, Carambola, Carrot, Celery, Cherry, Chili, Cucumber, Black Currant, Red Currant, Dandelion leaves, Fennel, Fig, Grapefruit, Kaki, Kale, Kiwi, Kumquat, Lemon, Lettuce, Mandarin, Mango, Melon, Orange, Papaya, Passion Fruit, Peach, Pear, Pepper, Pineapple, Pomegranate, Potato, Radish, Rambutan,, Raspberry, Spinach, Onion, Strawberry, Tomato, Watercress	$SnCl_2$, HCl, $Cu(CH_3COO)_2$, Diethanolamine	None	UV-Vis (372, 430 nm)	4404	60-120%	0.1-3.25	242 (>0.1 mg/kg)	18	0.05 (Banana, Beetroot, Kumquate, Papaya, Passion Fruit, Spinach, Raspberry) 0.5 (Cucumber) 1 (Beans) 2 (Apricot, Chili)	Denmark	2003	Poulsen et al.	Danish Veterinary and Food Administration, Soborg	DOI: 10.1080/0265203031000152433	Juhler Method (1999)	European Union, 1997a, Commission Directive 97/71/EC of 15 December 1997 amending the Annexes to Council Directives 86/362/EEC, 86/363/EEC and 90/642/EEC on the fixing of maximum levels for pesticide residues in and on cereals, foodstuffs of animal origin and certain products of plant origin, including fruit and vegetables respectively.
13	None	Tomato, Plum, Strawberry, Currant, Lettuce, Mushrooms, Cherry, Cucumber, Apple	HCl; $SnCl_2$; KOH; Methanol, $Zn(CH_3COO)_2$; p-Dimehtylamino aniline; Iron (III)	None	Vis (662 nm)	5583	N.A.	N.A.	717 (>0.05 mg/kg for Mushrooms, >0.2 mg/kg for Lettuce, >0.1 mg/kg for others)	146	0.05 (Mushrooms) 0.5 (Cucumber) 1 (Cherry, Plum) 2 (Strawberry) 3 (Tomato) 5 (Lettuce, Currant)	Poland	2003	Nowacka A.	Institute of Plant Protection, Regional Experimental Station Białystok	ISBN: 961-90950-2-2	Xanthate Method	MRL by Council Directives 86/362/EEC and 90/642/EEC with amendments up to 2001/57/EC
14	Mancozeb<br>Ziram	Apple, Banana, Orange, Papaya, Strawberry, Potato, Tomato, Dry Beans, Rice	HCl; $SnCl_2$; $Cu(CH_3COO)_2$	None	UV-Vis (435 nm)	520	>70%	0.13-3.8	316 (>0.10 mg/kg)	N.A.	0.2 (Strawberry) 0.3 (Dry Beans, Potato)	Brazil	2004	Caldas et al.	Department of Pharmaceutical Sciences, College of Health	DOI: 10.1016/j.fct.2004.07.006	Caldas Method (Improvement to Cullen and	ANVISA, 2004. Agencia Nacional de Vigilancia Sanitaria.

	Thiram										1 (Banana) 2 (Apple, Tomato, Orange) 3 (Papaya, Rice)				Sciences and Chemistry Institute, University of Brasilia		Keppel Methods)	Toxicologia. Available from: http://www.anvisa.gov.br/toxicologia/monografias/index.htm
15	Ziram	Courgette	$SnCl_2$, HCl, Isooctane	GC	MS	N.A.	>88%	0.03-5.0	Spiked Samples (> 0.03 mg/kg)	0	N.A.	Italy	2004	Guidotti M. et al.	ARPA, Regional Agency for Environmental Prevention, Abruzzo	N.A.	Validation of GC-MS Method	N.A.
16	Carbon Disulfide	Raspberry	Isooctane, HCl, $SnCl_2$	GC	ECD	N.R.	N.R.	0.07-0.23	N.R. (>0.05 mg/kg)	N.A.	N.A.	Serbia	2004	Kovacevic et al.	Faculty of Agriculture, Zemun-Belgrade	UDK: 634.711:632.95.028	No MRL provided in Yugoslavia for Dithiocarbamates in Raspberry at that time	Official Gazette of the Federal Republic of Yugoslavia, 1992
17	Maneb	Tomato	Isooctane, HCl, $SnCl_2$	GC	FPD	N.A.	70-113%	N.R.	N.R. (>0.03 mg/kg)	N.A.	3 (Tomato)	Greece	2004	Kontou et al.	Laboratory of Food Chemistry and Technology, School of Chemical Engineering, National Technical University of Athens	DOI: 10.1080/02652030400019372	ETU is a toxic metabolite of Maneb and in this article Maneb was analyzed through GC-MS after the conversion in CS_2 and ETU in HPLC-PDA	Commission Directive 98/82/EC
18	Carbon Disulfide	Chinese Chive, Carrot, Spinach, Lettuce, Grape, Chinese Mustard, Celery, Radish, Ching Geeng, Wax apples, Cabbage, Bitter melon, Green Pepper	$SnCl_2 \cdot 2H_2O$, HCl	GC	FPD	1999	N.A.	0.1-2.9	34 (>0.1 mg/kg)	0	0.5 (Radish) 2.5 (Bitter melon, Chinese Cabbage, Green Pepper) 4 (Celery, Chinese Mustard) 5 (Wax Apples)	Taiwan	2005	Chang et al.	Central Region Laboratory, Bureau of Food and Drug Analysis, Department of Health, Taichung City	DOI: 10.38212/2224-6614.2563		Taiwan Food and Drug Administration. (2005). Standards for pesticide residue limits in foods. Ministry of Health and Welfare, Taiwan.

19	N.R.	Apple, Lettuce, Potato	Isooctane, HCl, $SnCl_2$	GC	MS	404	N.R.	0.05-6.36	134 (>0.05 mg/kg)	37	0.1 (Potato) 3.0 (Apple, Tomato) 5.0 (Lettuce)	Slovenia	2006	Česnik et al.	Agricultural Institute of Slovenia, Central Laboratories, Ljubljana	DOI: 10.1080/02652030500401199	Method EURL	Official Gazette of the Republic of Slovenia No. 84, 30.07.2004, pp. 10210–10226.
20	None	Apple, Tomato, Papaya, Lettuce, Strawberry, Banana, Orange, Carrot, Potato, Beans, Rice	HCl; $SnCl_2$; $Cu(CH_3COO)_2$	None	UV-Vis (435 nm)	3821	N.A.	>0.05	1018 (>0.05 mg/kg for Orange, >0.1 mg/kg for Beans and Rice, >0.08 mg/kg for others)	99	0.2 (Strawberry) 0.3 (Potato) 2 (Apple) 6 (Lettuce)	Brazil	2006	Caldas et al.	Department of Pharmaceutical Sciences, College of Health Sciences and Chemistry Institute, University of Brasilia	DOI: 10.1016/j.fct.2006.04.014	Caldas Method (Improvement to Cullen and Keppel Methods)	ANVISA, 2005. Agencia Nacional de Vigilancia Sanitaria. Ministerio da Saude. Monografias de Produtos Agrotóxicos. Available from: <http://www.anvisa.gov.br/toxicologia/monografias/monografias.pdf
21	Thiram	Apple, Lettuce, Potato, Strawberry, Tomato	Isooctane, HCl, $SnCl_2$	GC	MS	150	96-111%	0.1-5.0	N.A. (>0.013 mg/kg)	N.A.	0.1 (Potato) 2.0 (Strawberry) 3.0 (Apple, Tomato) 5.0 (Lettuce)	Slovenia	2006	Česnik et al.	Agricultural Institute of Slovenia, Central Laboratories, Ljubljana	URN:NBN:SI:doc-3SGXNISC	Method EURL	Official Gazette of the Republic of Slovenia No. 84, 30.07.2004, pp. 10210–10226.
22	Thiram<br>Mancozeb	Peach, Green Beans, Apple, Tomato, Green Pepper, Potato, Fruit Cream Powder, Dehydrated vegetable cub	Isooctane, HCl, $SnCl_2$	GC	FPD	12	81-113%	0.01-0.1	N.A.	N.A.	N.A.	Greece	2006	Papadopoulou-Mourkidou et al.	Aristotle University of Thessaloniki	ISBN 1-58829-410-2	Method EURL	N.A.
23	None	Apples, Cauliflower, Cereals, Grapes, Lettuce, Peas, Peeper, Potato, Strawberries	Isooctane, HCl, $SnCl_2$	GC	MS	181	N.A.	>0.05	41 (>0.05 mg/kg for all Commodities)	0	N.A.	Slovenia	2007	Česnik et al.	Agricultural Institute of Slovenia, Central Laboratories, Ljubljana	URL: https://jcea.agr.hr/issues/article/778	Method EURL	Official Gazette of the Republic of Slovenia No. 84, 30.07.2004, pp. 10210–10226.
24	Ziram	Apple, Pear, Cherry, Grape,	Isooctane, HCl, $SnCl_2$	GC	MS	12	N.R.	N.R.	7 (>0.05 mg/kg)	1	0.05 (Tamarillos)	Germany	2008		Institute of Food		Method EURL	Ruckstands-Hochstmengen

	Carbon Disulfide<br>Dithiane<br>Antracol	Tomato, Cucumber, Tamarillos, Papaya, Broccoli												Crnogorac et al.	Chemistry, University of Hohenheim	DOI: 10.1016/j.trac.2008.10.008		verordnung (RHmV), in the version of publication of 21.10.1999 (Bundesgesetzblatt I p. 2082), last change 8.10.2007 (Bundesgesetzblatt I p. 2379)
25	Mancozeb<br>Mefenoxam	Green Beans	NaOK, KI, $SnCl_2$, HCl, Ethanol	None	UV-Vis (435 nm)	215	N.A.	0.05-2.65	43 (>0.05 mg/kg)	4	1 (Green Beans)	Morocco	2009	Bazzi et al.	Energy and Environmental Process Engineering Laboratory, National School of Applied Sciences, Agadir	URL:https://www.proquest.com/scholarly-journals/pesticide-residue-monitoring-green-beans-souss/docview/2122418495/se-2?accountid=27662	Cullen Method	Directive Nº2005/396/CE, February 23, 2005. EU Official Bulletin
26	None	Fig, Mango, Papaya, Persimmon	$SnCl_2$, HCl, $Cu(CH_3COO)_2$, Diethanolamine	None	UV-Vis (435 nm)	112	N.A.	0.5-1.0	17 (>0.01 mg/kg for Fig, Mango, Papaya, Persimmon)	12	0.05 (Fig, Persimmon) 2.0 (Mango) 7.0 (Papaya)	Brazil	2009	Ciscato et al.	Biological institute, Av. Conselheiro Rodrigues Alves, Sao Paulo	DOI: 10.1080/19440040903330326	Caldas Method	N.A.
27	None	Tomato, Cabbage, Lettuce, Cucumber, Carrot, Spinach, Potato, Onion, Pepper	Isooctane, HCl, $SnCl_2$	GC	ECD	145	N.A.	N.R.	21 (>0.01 mg/kg)	2	1 (Onion, Cabbage) 2 (Cucumber) 3 (Tomato)	Serbia	2009	Lazic et al.	Faculty of Agriculture, Novi Sad	DOI: 10.17660/ActaHortic.2009.830.82	Method EURL	EC 2008. Commission Regulation 149/2008/EC, Official Journal, L 58/1.
28	Thiram<br>Zineb<br>Ziram	Tangerine, Clementine, Orange, Peach, Nectarine, Khakis	Isooctane, HCl, $SnCl_2$	GC	MS	429	75-105%	0.006-0.12	37 (>0.5 mg/kg)	0	3 (Peach, Orange, Khakis)	Spain	2009	Berrada et al.	Laboratory of Food Chemistry and Toxicology, Faculty of Pharmacy, University of Valencia	DOI: 10.1016/j.foodcont.2009.03.011		EC 2008. Commission Regulation 149/2008/EC, Official Journal, L 58/1.
29	Maneb	Grape, Strawberry, Carrot, Lettuce, Corn	EDTA, NaOH, $SnCl_2*2H_2O$	VP-LPME	IR	N.A.	83-103%	0.011-0.036	Spiked Samples (0.06-	N.A.	N.A.	Spain	2010	Gonzalvez et al.	Department of Analytical Chemistry,	DOI: 10.1016/j.aca.2010.12.037	Validation of a New Method	N.A.

			, Tetrachloroethylene						0.120 mg/kg)						Research Building, University of Valencia			
	Ziram			GC	MS		N.A.	N.A.	N.A.									
	Mancozeb																	
30	Carbon Disulfide	Grape Leaf, Lettuce, Cantaloupe, Cucumber, Eggplant, Green Beans, Green Peas, Pepper, Tomato, Apple, Grape, Peach, Strawberry, Squash, Broccoli, Potato, Apricot, Orange, Plum, Watermelon	Ethanol, Diethanolamine, HCl, Toluene, Na_2SO_4, NaOH, $SnCl_2 \cdot 2H_2O$	None	UV-Vis (435 nm)	535	80-110%	0.11-5.09	122 (> 0.1 mg/kg)	7	0.2 (Potato) 0.5 (Cantaloupe, Onion) 1 (Pepper, Squash, Watermelon) 2 (Apple, Cucumber, Green Beans, Orange, Tomato) 5 (Grape, Broccoli, Strawberry) 7 (Apricot, Peach, Plum) 10 (Lettuce, Grape Leaf)	Egypt	2010	Khorshed et al.	Central Laboratory of Residue Analysis of Pesticides and Heavy Metals in Food, Agricultural Research Center, Ministry of Agriculture and Land Reclamation	DOI:10.21608/jppp.2010.86931	Cullen Method	Codex Alimentarius Commission (2005). GEMS/FOOD Cluster Diets, 2006 (WHO/FSF/FOS/98.3) Global Environment Monitoring System / Food Contamination Monitoring and Assessment Programme (GEMS/FOOD) World Health Organization
	Sodium Diethyl DTC																	
31	Carbon Disulfide	Green Beans, Green Peas, Broccoli, Green Onion, Potato Leaves, Peanut	Ethanol, Diethanolamine, HCl, Toluene, Na_2SO_4, NaOH, $SnCl_2 \cdot 2H_2O$	GC	MS	71	84-104%	0.05-5.0	7 (> 0.05 mg/kg)	2	0.1 (Peanut) 0.2 (Green Peas) 0.3 (Potato, Leaves) 1.0 (Green Beans, Green Onion, Broccoli)	Egypt	2011	El-Gohary et al.	Central Laboratory of Residue Analysis of Pesticides and Heavy Metals in Food, Agricultural Research Center, Ministry of Agriculture and Land Reclamation	DOI: 10.21608/jppp.2011.84656	Cullen Method	Codex Alimentarius Commission (2005). GEMS/FOOD Cluster Diets, 2006 (WHO/FSF/FOS/98.3) Global Environment Monitoring System / Food Contamination Monitoring and Assessment Programme (GEMS/FOOD) World Health Organization
	Sodium Diethyl DTC																	

32	None	Apple	Ethanol, Diethanolamine, HCl, Toluene, Na_2SO_4, NaOH, $SnCl_2 \cdot 2H_2O$	None	UV-Vis (435 nm)	212	N.A.	0.05–0.57	57 (> 0.1 mg/kg)	0	5 (Apple)	Poland	2011	Łozowicka et al.	Institute of Plant Protection, National Research Institute, Pesticide Residue Laboratory, Białystok	PL ISSN: 2083-4772	Cullen Method	European Parliament and European Commission Regulation issued on the 23 of February 2005 no. 396/2005 with subsequent changes (Dz. U. L 70 from 16 of March 2005) regarding the highest matter (2005)
33	Thiram	Apple, Leek, Potato, Strawberry, Wheat, Tomato, Lettuce, Rice	$SnCl_2$, HCl, Isooctane	GC	FPD	150	71-112%	0.1-10	12 (>0.1 mg/kg)	0	1 (Potato, Wheat)<br>3 (Leek, Tomato)<br>5 (Apple)<br>10 (Strawberry)	Greece	2012	Bempelou et al.	Laboratory of Pesticide Residues, National Reference Laboratory, Department of Pesticides Control and Phytopharmacy, Kifissia (Athens)	DOI:10.5555/20123271139	Validation of GC-FPD method for Bromide and CS_2	Regulation (EC) No396/2005 of European Parliament and of the council of 23 February 2005. On maximum residue levels of pesticides in or on food and feed of plant and animal origin and amending Council Directive 91/414/EEC. L 70, 16.3.2005.
34	Carbon Disulfide<br>Sodium Diethyl DTC	Watermelon, Banana, Mango, Cauliflower, Potato, Apricot, Grape, Green Peas, Lettuce, Molokhia, Watercress, Cucumber, Eggplant, Squash, Tomato,	Ethanol, Diethanolamine, HCl, Toluene, Na_2SO_4, NaOH, $SnCl_2 \cdot 2H_2O$	None	UV-Vis (435 nm)	225	80-110%	0.15-5.1	77 (> 0.1 mg/kg)	2	0.2 (Potato)<br>0.5 (Cantaloupe, Onion)<br>1 (Pepper, Squash, Watermelon)<br>2 (Apple, Cucumber,	Egypt	2012	El-Sawi Sanaa A. et al.	Central Laboratory of Residue Analysis of Pesticides and Heavy Metals in Food, Agricultural Research Center,	DOI: 10.21608/jppp.2012.83759	Method EURL	Codex Alimentarius Commission (2005). GEMS/FOOD Cluster Diets, 2006 (WHO/FSF/FOS/98.3) Global Environment

		Cantaloupe, Guava, Strawberry, Spinach, Grape Leaf									Green Beans, Orange, Tomato) 5 (Grape, Broccoli, Strawberry) 7 (Apricot, Peach, Plum) 10 (Lettuce, Grape Leaf)				Ministry of Agriculture and Land Reclamation			Monitoring System / Food Contamination Monitoring and Assessment Programme (GEMS/FOOD) World Health Organization
35	Mancozeb	Raspberry	$SnCl_2$, HCl, Isooctane	GC	MS	103	>91%	0.03–0.8	81 (> 0.03 mg/kg)	21	0.05 (Raspberry)	Serbia	2012	Pucarevic et al.	Educons University, Faculty for Environmental Governance and Corporate Responsibility, Sremska Kamenica	DOI: 10.17660/ActaHortic.2012.946.54	Method EURL	COMMISSION REGULATION (EU) No 37/2010 of 22 December 2009 on pharmacologically active substances and their classification regarding maximum residue limits in foodstuffs of animal origin
36	Carbon Disulfide	Tomato, Paprika, Cucumber, Potato, Onion, Carrot, Cabbage, Ketchup, Apple, Cherry, Grape, Wine	$SnCl_2$, HCl, Isooctane	GC	MS	168	N.A.	N.A.	N.A.	N.A.	N.A.	North Macedonia	2014	Kostik et al.	Faculty of Medical Science, Department of Pharmacy, University "Goce Delchev", Shtip	DOI: 10.11648/j.jfns.20140204.15	The document doesn't explicitly report results about CS_2 quantification in samples	Regulation (EC) No 396/2005 of the European Parliament and of the Council of 23 February 2005 on maximum residue levels of pesticides in or on food and feed of plant and animal origin and amending Council Directive 91/414/EEC

37	Mancozeb	Grape, Green Chili, Tomato, Potato, Brinjal, Pineapple, Chayote	$SnCl_2$, HCl, Isooctane	GC	MS	25	75-104%	0.04-1.49	7 (>0.04 mg/kg)	0	0.05 (Pineapple) 0.3 (Potato) 3 (Tomato) 5 (Grape)	India	2014	Mujawar et al.	National Referral Laboratory, National Research Centre for Grapes, Pune	DOI: 10.1016/j.foodchem.2013.10.148	Method EURL	Commission Reg. (EU) No 34/2013 of 16 Jan. 2013
38	None	Apple, Blueberry, Currant, Raspberry, Tomato, Broccoli, Parsley, Cucumber, Cabbage	Ethanol, Diethanolamine, HCl, Toluene, Na_2SO_4, NaOH, $SnCl_2 \cdot 2H_2O$	None	UV-Vis (435 nm)	317	N.A.	0.05-0.64	14 (>0.05 mg/kg)	0	2 (Apricot, Cherry) 3 (Tomato) 5 (Apple, Currant, Gooseberry, Grape, Pear)	Poland	2015	Szpykra E. et al.	Institute of Plant Protection, National Research Institute, Regional Experimental Station in Rzeszów	PMID: 26024398	Method EURL	Commission Regulation (EC) No 396/2005 of 23 February 2005 on maximum residue levels of pesticides in or on food and feed of plant and animal origin and amending Council Directive 91/414/EEC. Off J Eur Union L 70/1, 16.3.2005, with later amendments.
39	None	Apple, Black Berries, Chokeberries, Blueberries, Currants, Elderberries, Gooseberries, Pears, Plums, Raspberries, Sea Sallowthorns, Cherries, Strawberries	HCl; $SnCl_2$; KOH; Methanol, $Zn(CH_3COO)_2$; p-Dimehtylamino aniline; Iron (III)	None	Vis (662 nm)	123	N.A.	0.05–2.08	51 (> 0.02 mg/kg)	16	5 (Apple, Gooseberries, Currant) 10 (Strawberry)	Poland	2015	Łozowicka et al.	Institute of Plant Protection, National Research Institute, Pesticide Residue Laboratory, Białystok	DOI: 10.1515/jppr-2015-0018	Xanthate Method	Regulation (EC) No 396/2005 of the European Parliament and of the Council of 23 February 2005 on maximum residue levels of pesticides in or on food and feed of plant and animal origin and amending Council Directive 91/414/EEC as

																		follows changes.
40	None	Apple, Apricot, Black Currant, Gooseberry, Grape, Peach, Raspberry, Red Currant, Strawberry, Sweet Cherry, Broccoli, Brussels Sprout, Carrot, Celeriac, Dill, Lettuce, Parsley, Peaking Cabbage, Spinach, Tomato, Wheat	Ethanol, Diethanolamine, HCl, Toluene, Na_2SO_4, NaOH, $SnCl_2 \cdot 2H_2O$	None	UV-Vis (435 nm)	328	N.A.	0.05-3.60	5 (>0.05 mg/kg)	1	0.05 (Spinach) 2.0 (Apricot) 5.0 (Black Currant, Grape, Red Currant) 10.0 (Strawberry)	Poland	2016	Szpykra E. et al.	Institute of Plant Protection, National Research Institute, Regional Experimental Station in Rzeszów	ISSN: 0035-7715 eISSN: 2451-2311		Commission Regulation (EC) No 396/2005 of 23 February 2005 on maximum residue levels of pesticides in or on food and feed of plant and animal origin and amending Council Directive 91/414/EEC. Off J Eur Union L 70/1, 16.3.2005, with later amendments.
41	Mancozeb	Tomato	KI, $SnCl_2$, HCl, Distilled Water, NaOH, Diethanolamine, Ethanol, $SnCl_2 \cdot 2H_2O$	None	UV-Vis (435 nm)	90	86-94%	0.1-1	90 (>0.03 mg/kg)	0	N.A.	Morocco	2017	El Habib Ait Addi et al.	Engineering and Chemical Engineering Group, High School of Technology, University Ibn Zohr, Agadir	DOI: 10.21013/jas.v7.n2.p2	Cullen Method: The article investigates the dissipation Behavior of Mancozeb (as CS_2) in open field	N.A.

42	Mancozeb	Tomato	$SnCl_2$, HCl, Isooctane	GC	MS	225	N.A.	0.35-7.7	166 (N.A.)	18	2 (Tomato)	Uganda	2017	Atuhaire et al.	Uganda National Association of Community and Occupational Health (UNACOH), Kampala	DOI: 10.1177/1178630217712218	Method EURL	World Health Organization (WHO), Food and Agriculture Organization (FAO). Maximum Residue Limits for Dithiocarbamates in Tomato. Codex Alimentarius International Food Standards. http://www.fao.org/fao-who-codexalimentarius/ standards/pestres/pesticide-detail/en/?p_id=105. Published 2016.
43	Mancozeb	Leafy Vegetables (Lettuce, Chard, Spinach)	HCl; $SnCl_2$; KOH; Methanol, $Zn(CH_3COO)_2$; Ethylenediamine	None	UV (240-360 nm)	118	91.8%	0.34-40	9 (>0.01 mg/kg)	9	0.05 (Leaf Vegetables)	Chile	2017	Elgueta et al.	Department of Environment and Sustainability, Instituto de Investigaciones Agropecuarias, Santiago	DOI: 10.1080/19393210.2017.1280540	Perz Method (2000)	CODEX Alimentarius Commission 2011. [Internet]. [cited 2016 April 15]. Available from: http://www.fao.org/fao-whocodexalimentarius/ standards/list-of-standards/en/Accessed 15.4.16.
44	Thiram	Lettuce	$SnCl_2$, HCl, Isooctane, Diethanolamine, Ethanol, $SnCl_2*2H_2O$	GC	ECD	N.A.	88.9-107.4%	0.05-0.4	N.A.	N.A.	N.A.	Brazil	2017	Pizzutti et al.	Center for Research and Analysis of Residues and Contaminants (CEPARC),	DOI: 10.21577/0103-5053.20160227	Comparison of four different methods, 3 chromatographic	N.A.
				GC	PFPD		92.8-93.9%	0.05-0.4										
				GC	MS		94.0-97.4%	0.05-0.4										

				None	UV-Vis (435 nm)		87.7-94.0%	0.4-8.9							Department of Chemistry, Federal University of Santa Maria, Brazil		methods and classic spectrophotometric. Cullen extraction performed for UV-Vis determination and Iso-octane extraction after acid hydrolysis for GC.	
45	Mancozeb	Rice, Corn, Cabbage	Water, Ascorbic Acid, Hexane, $SnCl_2$, HCl,	GC	MS	50	75.7-91.0%	0.03-0.27	1 (>0.03 mg/kg)	0	N.A.	China	2017	Shao et al.	Tianjin Institute of Agricultural Quality Standard and Testing Technology, Ministry of Agriculture Lab of Agricultural Product Quality Safety Risk Assesment, Tianjin	ISSN: 2095-0381 CABI: 20173294167	New GC-MS method in which Hexane is acting as adsorber for CS_2 (gas) freed by the acid hydrolysis	In China, only limit for Apples was defined at that time (2016)
46	Propineb and metabolites (PDA-PTU)<br>Carbon Disulfide	Banana	$SnCl_2$, HCl, n-Hexane	GC	FPD	70	75.3-115.4%	0.01-177.4	70 (>0.01 mg/kg)	N.A.	N.A.	China	2017	Song et al.	Institute for Pesticide and Environmental Toxicology, Guangxi Key Laboratory Cultivation Base of Agro-Environment and Agro-Product	DOI: 10.1080/03601234.2017.1399765	The analysis of confirmation of metabolites for Propineb was performed using a LC-MS/MS. The study was mostly focused on the fate of Propineb and its	N.A.

																	metabolites in Bananas and Soil.	
47	Carbon Disulfide	Apricot	$SnCl_2$, HCl, Isooctane	GC	MS	20	N.A.	0.03-1.10	20 (>0.03 mg/kg)	N.A.	N.A.	Turkey	2018	Arslan et al.	Food Enterprises and Codex department, Ministry of Agriculture and Forestry, Ankara	DOI: 10.1080/19440049.2018.1562235	The aim of this study was to evaluate the effect of sulfur natural occurrence, giving false positives for CS_2. Cullen method is used.	N.A.
48	Zineb	Eggplant, Broccoli, Potato, Pear, Onion, Cabbage, Lettuce, Spinach, Lettuce, Ginger, Pepper, Cucumber, Cowpea, Tomato, Orange, Pumpkin, Strawberry, Banana, Papaya, Guava, Star Fruit, Watermelon, Apple, Radish, Carrot	EDTA, $SnCl_2$, HCl, Assisted Microwave Dystem	LC	DBD-MES	20	83.5-104%	4.65-25.0	20 (>0.01 mg/kg)	N.A.	N.A.	China	2018	Han et al.	Analysis & Testing Center, Chinese Academy of Tropical Agricultural Sciences, Hainan Provincial Key Laboratory of Quality and Safety for Tropical Fruits and Vegetables, Haikou	DOI: 10.1039/C8AN00613J	Developed of new Microwave-assisted stannous Chloride Hydrolysis with dielectric barrier discharge-carbon molecular emission spectrometry	N.A.
49	Carbon Disulfide	Tomato, Mango, Cabbage, Grape	$SnCl_2$, HCl, Isooctane	GC	ECD	12	84.9-98.7%	0.08-0.11	2 (>0.05 mg/kg)	N.A.	N.A.	Vietnam	2018	Nguyen et al.	Institute of Environmental Science, Engineering and Management, Southern pesticide control and testing Center	DOI: 10.46242/jst-iuh.v36i06.863	Development of new GC-ECD method for CS_2	N.A.
50	Thiram	Passion Fruit	HCl; $SnCl_2$; $Cu(CH_3COO)_2$,	None	UV-Vis (435 nm)	108	71-120%	0.06-0.7	37 (>0.05 mg/kg)	0	1 (Passion Fruit)	Brazil	2019	Mozzaquatro et al.	Department of Pharmaceutical Sciences,	DOI: 10.21577/0103-5053.20190091	Caldas Method,	Agência Nacional de Vigilância

			Diethanolamine, Ethanol, NaOH												College of Health Sciences and Chemistry Institute, University of Brasilia		vertical system	Sanitária (ANVISA), http://portal.anvisa.gov.br/documents/111215/117782/M02%2B%2BMancozebe.pdf/975fdd18-65fd-477c-ab85-217bcb9a0110
51	Mancozeb	Onion, Onion Leaves	HCl, $SnCl_2$, Ethyl acetate, Isooctane	GC	MS	16	84.47-97.16%	0.23-5.89	16 (>0.05 mg/kg)	N.A.	N.A.	India	2019	Patil et al.	AINP on Pesticide Residues, Department of Agril. Entomology, Ahmednagar, Maharashtra	DOI: 10.22271/chemi.2020.v8.i1ak.8630	The study was focused on the mancozeb dissipation in onions at 3, 5, 7 and 10 days	N.A.
52	Carbon Disulfide	Apple, Avocado, Papaya, Durian, Soursop, Lemon, Guava, Mango, Orange, Mangosteen, Passion Fruit, Pineapple, Pomelo, Banana, Pitaya, Rambutan, Rockmelon, Salacca, Watermelon, Jackfruit	HCl, $SnCl_2$	GC	FPD	176	N.A.	N.A.	N.A.	0	N.A.	Malaysia	2019	Rahman Alinah A.	Agriculture Research Centre Tuaran, Sabah Department of Agriculture, Tuaran, Sabah	URL: https://www.itfnet.org/istf2019/fp/P02.pdf	Pesticide Residue Monitoring for Farm accreditation in Malaysia	N.A.
53	Mancozeb	Lettuce	None	None	Vis-NIR (600 μm)	70	N.A.	1.41-10.3	N.A. (>1.41 mg/kg)	N.A.	N.A.	Brazil	2020	Steidle Neto et al.	Federal University of Sao Joao del-Rei, Campus Sete Lagoas, Minas Gerais	DOI: 10.13128/ahsc8125	New method developed in reflectance for Mancozeb	N.A.
54	Thiram	Soybean	$SnCl_2$, HCl, Isooctane	GC	ITD-MS<br>PFPD	N.A.	68-91%	0.05-0.5	(>0.05 mg/kg)	N.A.	N.A.	Brazil	2021	da Silva et al.	Chemistry Department, Center of Research and Analysis of Residues and Contaminants	DOI: L90:O90+U92	Validation of New Method	N.A.

															(CEPARC), Federal University of Santa Maria			
55	None	Arugula, Bean pod, Bean root, Carrot, Chayote, Chicory, Chili, Coriander, Chive, Eggplant, Ginger Leek, Lettuce, Parsley, Pumpkin, Scarlet eggplant, Spinach, Sweet pepper, Sweet potato, Tomato, Watercress, Yam, Zucchini	NaOH, Diethanolamine, Ethanol, $Cu(CH_3COO)_2$, $SnCl_2$, HCl	None	UV-Vis (435 nm)	84	N.A.	0.06-0.50	21 (>0.05 mg/kg)	0	0.1 (Potato, Tomato, Carrot) 0.3 (Bean pod, Bean root) 0.5 (Eggplant) 1 (Pumpkin, Zucchini) 3 (Lettuce)	Brazil	2022	de Araujo et al.	University of Brasilia, Laboratory of Toxicology, Pharmaceutical Sciences Department	DOI: 10.1590/s0102-0536-20220213	Caldas Vertical System	ANVISA (Agência Nacional de Vigilância Sanitária). 2021. Panel of authorized monographs. Available at: http://portal.anvisa. gov.br/registros-e-autorizacoes/agrotoxicos/ produtos/monografia-de agrotoxicos. Accessed on December 20, 2021
56	Carbon Disulfide	Vine Leaves	$SnCl_2$, HCl, Ethanol, Diethanolamine, $SnCl_2 \cdot H_2O$	GC	MS	10	74-107%	0.05-0.2	10(>0.04 mg/kg)	10	0.05 (Vine Leaves)	Turkey	2022	Arslan et al.	Ministry of Agriculture and Forestry, General Directorate of Food and Control, Ankara	DOI: 10.1080/19440049. 2022.2093987	Study on Vine Leaves potential false positives due to natural occurring Sulfur in CS_2 determination	Codex Alimentarius 2022b. Codex pesticides residues in food online database. https://www.fao.org/fao-who-codexalimentarius/ codex-texts/dbs/pestres/pesticides/en/.
57	Carbon Disulfide	Cardamom, Black Pepper	$SnCl_2$, HCl, Isooctane	GC	MS	12	75-98%	0.05-1.0	N.A. (>0.05 mg/kg)	N.A.	0.1 (Cardamom, Black Pepper)	India	2022	Natarajan et al.	Quality Evaluation Laboratory, Spices Board, Palarivattom, Kochi	DOI: 10.1007/s13197-022-05462-9	Validation study for CS_2 on Cardamom and Black Pepper	Codex MRL, Cardamom and Pepper (2015) Codex Maximum Residue Limits in HS 0775 - Cardamom.

																		http://www.fao.org/fao-who-codexalimentarius/codex-texts/dbs/pestres/commodities-detail/en/?lang=en&c_id=745.
58	Thiram	Yerba Mate	$SnCl_2$, HCl, Isooctane, GCB, $MgSO_4$	GC	MS	65	71.3-118.2%	0.14-0.79	6 (>0.1 mg/kg)	6	0.1 (Mate Tea)	Brazil	2022	da Silva et al.	Chemistry Department, Center of Research and Analysis of Residues and Contaminants (CEPARC), Federal University of Santa Maria	DOI: 10.1016/j.foodchem.2022.133513	EURL modified method	European Commission. (2017). Pesticide residue(s) and maximum residue levels (mg/kg). Reg. (EU) 2017/171 of 30 January 2017.
59	Mancozeb<br>Metiram<br>Thiram<br>Propineb	Mango, Banana, Rice, Cowpea, Lychee, Cabbage	EDTA, $SnCl_2$, HCl, Assisted Microwave System	LC	LEGD-DBD-MES (257.94 nm)	3	73-119%	0.1-25	N.A.	N.A.	N.A.	China	2023	Tian et al.	Analysis & Testing Center, Chinese Academy of Tropical Agricultural Sciences, Hainan Provincial Key Laboratory of Quality and Safety for Tropical Fruits and Vegetables, Haikou	DOI: 1/0.1016/j.foodchem.2023.136884	EURL modified method	N.A.
60	Mancozeb	Banana, Mango, Pineapple, Cowpea, Dragon Fruit, Lychee, Apple, Eggplant, Peanuts	$SnCl_2$, HCl, Ascorbic Acid, NaOH, EDTA, L-Cysteine, Hexane, Assisted Microwave System	GC	ECD	50	81-112%	0.0811-9.86	0 (>0.01 mg/kg)	0	0.05 (Lowest MRL as reference)	China	2024	Tian et al.	Analysis & Testing Center, Chinese Academy of Tropical Agricultural Sciences, Hainan Provincial Key Laboratory of Quality and	DOI: 10.1155/2024/2577585	Development of one pot, microwave assisted extraction and acidolysis. Mancozeb Spikes at 0.01, 1.0	N.A.

															Safety for Tropical Fruits and Vegetables, Haikou		and 10 mg/kg	
**61**	Mancozeb	Cauliflower	H_2SO_4, $Cu(CH_3COO)_2 \cdot H_2O$, Diethanolamine, $Pb(CH_3COO)_2$	HPLC	MS-MS	30	70.42-129.14 %	0.017-0.180	24 (>0.01 mg/kg)	0	N.A.	India	2025	Tripathi et al.	Division of Agricultural Chemicals, Indian Agricultural Research Institute, New Delhi	DOI: 10.1016/j.jcoa.2025.100226	Development of new method for Mancozeb (as CS_2) detection in Cauliflower	N.A.

Table S2. Analytical methods published in years 2000-2025 for determination of dithiocarbamates without CS_2 formation, detailed information.

#	Target Compounds	Matrix	Reagents	Chromatographic Method	Detection Method	Samples Analyzed	Recovery Ranges	Ranges found (mg/kg)	Samples Above LOQ	Samples Above MRL	MLR (dithiocarbamates expressed as CS_2, mg/Kg)	Location	Year	Author	Leading Institute	Reference (DOI, PMID, ISBN, URL, Others)	Notes	Reference Regulation
**1**	Ziram	Potato, Cabbage, Tomato, Cucumber	Arsenazo (III), NaF, Sodium Citrate, Sodium Thiosulphate, Sodium Tartrate, $MnCl_2$, $CaCl_2$, MgCl, Diethanolamine	None	Vis (590 nm)	N.A.	90-106.5%	19.72-46.80	N.A. (> 19.72 mg/kg)	N.A.	N.A.	Malaysia	2001	Saad et al.	School of Chemical Science, Universiti Sains Malaysia, Penang	URL: https://www.ukm.my/mjas/v7_n1/17-bahruddin103.pdf	The linearity was studied in a range of 2,34-46.80 mg/kg of Ziram Spiked into food matrices, high interferences due to different metal ions until 19.72 mg/kg, thus considered as the LOQ.	N.A.
**2**	Ethylene thiourea	Tomato, Tomato Juice, Tomato Paste	Methanol, Water, NH4Cl, KF, Na_2SO_4, DCM	HPLC	PDA	N.R.	71.6-86.9%	0.01-0.5	N.A. (>0.01 mg/kg, Tomato Juice >0.05 mg/kg, Tomato Paste)	N.A.	N.A.	Greece	2001	Kontou et al.	Laboratory of Food Chemistry and Technology, School of Chemical Engineering, National	DOI: 10.1021/jf000116e	Development and validation of HPLC-PDA method for ETU, no MRL available for this metabolite	N.A.

															Technical University of Athens			
**3**	Dazomet<br>Disulfiram<br>Thiram<br>Ethylene thiourea<br>Propylene thiourea	Avocado, Cherry, Lemon, Nuts, Oat, Orange, Peach, Rice, Tomato	Distilled Water, DCM, Methanol	LC	APCI-MS	N.A.	33-109%	0.25-25	N.A. (> 0.25 mg/kg)	N.A.	N.A.	Spain	2003	Blasco et al.	Bromatology and Toxicology Laboratory, University of Valencia, Burjassot	DOI: 10.1016/j.chroma.2003.12.002	Method Validation for different dithiocarbamates in Fruit and vegetables	N.A.
**4**	Ethylene thiourea	Tomato	Methanol, Water, NH_4Cl, KF, Na_2SO_4, DCM	HPLC	PDA	N.A.	N.A.	N.R.	N.A. (> 0.002 mg/kg)	N.A.	N.A.	Greece	2004	Kontou et al.	Laboratory of Food Chemistry and Technology, School of Chemical Engineering, National Technical University of Athens	DOI: 10.1080/02652030400019372	ETU is a toxic metabolite of Maneb and in this article Maneb was analyzed through GC-MS after the conversion in CS_2 and ETU in HPLC-PDA	N.A.
**5**	Mancozeb<br>Propineb	Cucumber, Apple	Luminol, $K_3[Fe(CN)_6]$, $K_4[Fe(CN)_6]\cdot 3H_2O$, Water, EDTA, TCEP	HPLC	CL	N.R.	78.-109.2%	N.R.	N.R. (>0.0003 mg/kg, Mancozeb, >0.0014, Propineb)	N.A.	N.A.	Japan	2004	Nakazawa et al.	Department of Analytical Chemistry, Faculty of Pharmaceutical Sciences, Hoshi University, Tokyo	DOI: 10.1081/JLC-120028258	Validation of new method based on chemiluminescence in presence of Hexacyanoferrate	N.A.
**6**	Maneb	Tomato, Wheat Grain, Water	Water, HCl	None	FAAS	N.A.	95.5-97.5	2.4-24	N.A. (> 2 mg/kg)	N.A.	N.A.	Turkey	2005	Turker et al.	Gazi University, Science and Art Faculty,	ISSN: 1303-9709	Determination of Manganese coming from	N.A.

															Department of Chemistry, Ankara		Maneb degradation through FAAS	
**7**	Ziram<br>Dithiane<br>Antracol<br>Zineb<br>Propineb	Grapes, Cucumbers, Tomatoes, Rucola	$NaHCO_3$, DL-penicillamine, L-Cysteine, EDTA, NTA, Water	HPLC	MS	N.R.	90-100%	0.01-0.9	N.A. (>0.05 mg/kg)	N.A.	N.A.	Germany	2007	Crnogorac et al.	Institute of Food Chemistry, University of Hohenheim	DOI: 10.1002/rcm.3312	New Method Validation for analysis of three subclasses of DTC (DMDs, EBDs, PBDs)	N.A.
**8**	Ziram<br>Dithiane<br>Antracol	Apple, Pear, Cherry, Grape, Tomato, Cucumber, Tamarillos, Papaya, Broccoli	$NaHCO_3$, DL-penicillamine, L-Cysteine, EDTA, NTA, Water	HPLC	MS-MS	12	97-101%	0.005-1	7 (>0.005 mg/kg)	1	0.05 (Tamarillos)	Germany	2008	Crnogorac et al.	Institute of Food Chemistry, University of Hohenheim	DOI: 10.1002/rcm.3646	New Method Validation for analysis of three subclasses of DTC (DMDs, EBDs, PBDs) and comparison with classic CS_2 method	Ruckstands-Hochstmengenverordnung (RHmV), in the version of publication of 21.10.1999 (Bundesgesetzblatt I p. 2082), last change 8.10.2007 (Bundesgesetzblatt I p. 2379)
**9**	Mancozeb<br>Maneb<br>Zineb	Persimmons, Pears, Strawberries, Cabbage, Lettuce, Spinach	EDTA, Dimethyl Sulfate, Acetonitrile, $MgSO_4$, NaCl, L-Cysteine	HPLC	MS-MS	N.A.	71-101%	0.001-0.200	N.A.(>0.008 mg/kg)	N.A.	N.A.	Japan	2008	Hayama et al.	Environmental Science Center, Foundation for Kyushu Environmental and Occupational Health, Kurume, Fukuoka	DOI: 10.1007/s00216-008-2346-8	New method for EBDCs simultaneous determination after methylation	N.A.
**10**	Ziram	Potato, Cabbage, Tomato	HCl, Acetone, Ethanol	None	Square Wave Voltamm	3	93.3-97.8%	1.95-3.77	N.A. (>0.02 mg/kg)	N.A.	N.A.	China	2008	Qiu et al.	State Key Laboratory of Food	DOI: 10.1016/j.cclet.2008.07.013	Only 3 real samples	N.A.

					etry (SWV)										Science and Technology, Nanchang University		investigated	
**11**	Ziram<br>Thiram<br>Ferbam<br>Nickel bis(dithiocarbamate)<br>Propineb<br>Maneb<br>Zineb<br>Mancozeb<br>Polycarbamate<br>Milneb	Cacao, Spinach, Potato, Brown Rice, Pumpkin, Orange, Soybean, Cabbage, Apple, Green Tea	Cysteine, EDTA, CH_3I, DCM	GC	MS	N.R.	72-120%	0.01-0.2	N.A. (>0.1 mg/kg, Tea >0.01 for Others)	N.A.	0.02 (Cacao) 0.2 (Spinach, Potato) 0.3 (Brown Rice) 1 (Pumpkin) 2 (Orange) 3 (Soybean) 5 (Cabbage, Apple, Green Tea)	Japan	2010	Nakamura et al.	Japan Food Research Laboratories, Tokyo	DOI: 10.3358/shokueishi.51.213	DTCs are Extracted from Food with Cysteine-EDTA solution and Sodium salts, and subsequently methylated with CH_3I	Positive List System for Agricultural Chemical Residues in Foods Department of Food Safety, Ministry of Health, Labor and Welfare of Japan, June 2006
**12**	Ethylene thiourea<br>Propylene thiourea	Celery, Melon, Spinach	Methanol, Water	UHPLC	MS-MS	N.R.	65-127%	0.05-0.3	N.R. (>0.05 mg/kg)	N.A.	N.A.	Belgium	2011	Bonnechere et al.	Food, Medicine and Consumer Safety Department, Scientific Institute of Public Health, Ixelles	DOI: 10.1016/j.chroma.2011.04.083	Validation of rapid and environmentally friendly analytical method	N.A.
**13**	Thiram	Apple, Pear, Strawberry, Lettuce	Acetonitrile, $MgSO_4$, NaCl, PEG 600, Water	DART	MS	N.R.	94-102%	0.1-10	N.R. (>0.1 mg/kg, Thiram; >0.5 mg/kg, Ziram)	N.A.	N.A.	Czech Republic	2011	Cajka et al.	Department of Food Chemistry and Analysis, Faculty of Food and Biochemical Technology, Prague	DOI: 10.1080/19440049.2011.590456	Development of two method one for Thiram and Ziram (DART) and another for Ziram (DESI)	N.A.
	Ziram			DESI	MS-MS		70-85%		N.R. (>0.1 mg/kg, Thiram)									

**14**	Thiram	Tomato	EDTA, L-Cysteine, TBAHS, CH_3I, 1-2 propanediol, DCM, Hexane, HCl, NaCl, MeOH	HPLC	UV (272 nm)	80	92-104%	0.125-3.2	1 (>0.05 mg/kg, Thiram)	1	3 (Tomato)	Iran	2012	Jafari et al.	Department of Pharmacology, School of Medicine, Tehran	DOI: 10.1080/19393210.2012.657693	Monitoring of DTCs in Greenhouse and non-Greenhouse tomatoes	Codex Alimentarius. 2007. Report of the Thirty-ninth Session of the Codex Committee on Pesticide Residues. Beijing, China. 7–12 May 2007
	Mancozeb								2 (>0.20 mg/kg, Mancozeb)									
	Propineb								0 (>0.20 mg/kg, Propineb)									
**15**	Thiram	Eggplant, Lettuce, Strawberry, Apple	EDTA, Na_2SO_4, Acetonitrile, $NaHCO_3$	HPLC	MS-MS	N.R.	80-106%	0.01-0.13	N.A. (>0.01 mg/kg)	0	N.A.	Spain	2012	Peruga et al.	Research Institute for Pesticides and Water, University Jaume I, Castellon	DOI: 10.1016/j.foodchem.2012.04.064	Effect of Low temperature, addition of dehydrating agent, pH regulator and enzymatic activity reduction have been investigated	N.A.
**16**	Ethylene thiourea	Rice, Leaf Mustard	Na_2SO_4, NaCl, CH_3COOH, Acetonitrile	UPLC	MS-MS	600	97-116%	0.005-0.390	61 (>0.005 mg/kg)	0	N.A.	Hong Kong	2012	Chung et al.	Food Research Laboratory, Food and Environmental Hygiene Department, Kowloon	DOI: 10.1007/s00216-012-5882-1	No level set for ETU and i-PTU but the concentration found is far below the limit for dithiocarbamate set by Codex	N.A.
	Propylene thiourea							0.005-120										
**17**	Mancozeb	Apple, Wine Grape, Lettuce, Pepper, Tomato, Strawberry	L-Cysteine, EDTA-2Na,	HPLC	UV (270 nm)	150	74-139%	0.02-22.5	91 (>0.11 mg/kg, Mancoz	9	5 (Peppers, Lettuce)	Spain	2012	Lopez-Fernandez et al.	Nutrition and Bromatology Group,	DOI: 10.1016/j.foodchem.2012.02.178	Monitoring of DTCs residues in Spain	EU (2010). Commission Regulation (EU) No. 750/2010 of

	Maneb		Dimethyl Sulfate Solution in Acetonitrile, Mg_2SO_4, NaCl				78-115%		eb; >0.12 mg/kg, Maneb; >0.02 mg/kg, Propineb)						Analytical and Food Chemistry Department, Faculty of Food Science and Technology, University of Vigo, Ourense			7 July 2010 amending annexes ii and iii to regulation (EC) No. 396/2005 of the European parliament and of the council as regards maximum residue levels for certain pesticides in or on certain products. Official Journal L 220, 21/08/2010 P. 0001–0056.
	Propineb						99-128%											
**18**	Antracol<br>Ferbam<br>Maneb<br>Mancozeb<br>Propineb<br>Thiram<br>Ziram	N.A.	Acetonitrile, Methanol, Ethanol, NaOH	MALDI	MS (Orbitrap)	N.A.	N.A.	0.045-200.11	N.A. (>0.15 mg/kg, for all analytes)	N.A.	N.A.	Germany	2013	Ivanova et al.	Department of Analytical Chemistry, Institute of Environmental Research, Faculty of Chemistry, University of Dortmund	DOI: 10.1007/s11356-013-1837-0	Based on determination of Chelant Transition metal	N.A.
**19**	Thiram	Tomato, Grape, Sweet Peppers, Nectarine, Peach	NaOH, $NaHCO_3$, Penicillamine Buffer, Na_2SO_3, H_2S2O_5	HPLC	MS-MS	N.A.	80-108%	0.02-1	N.A. (>0.002 mg/kg)	N.A.	N.A.	Germany	2013	Ringli et al.	Institute of Food Chemistry, University of Hohenheim	DOI: 10.1080/19440049.2013.833669	Validation of new hydrophilic interaction LC-MS method	N.A.
**20**	Mancozeb	N.A.		None	Adsorptive	N.A.	81.8-97.6%	0.005-0.750	N.A. (>0.010	N.A.	N.A.	Italy	2013	Amorello et al.	Biologic, Chemistr			N.A.

			Na_2EDTA, Acetone, Na_2HPO_4		Stripping Voltammetry (AdSV)				mg/kg, Mancozeb)						y and Pharmaceutical Technology and Science Department, University of Palermo	DOI: 10.1016/j.microc.2013.05.002	Micro-determination of dithiocarbamates in pesticide formulations using voltammetry	
	Maneb								N.A. (>0.0062 mg/kg, Maneb)									
	Propineb								N.A. (>0.022 mg/kg, Propineb)									
	Nabam								N.A. (>0.048 mg/kg, Nabam)									
	Na $(CH_3)_2DTC$								N.A. (>0.018 mg/kg, Na$(CH_3)_2$DTC)									
	Zineb								N.A. (>0.022 mg/kg, Zineb)									
	Ziram								N.A. (>0.038 mg/kg, Ziram)									
	Ferbam								N.A. (>0.037 mg/kg, Ferbam)									
	Thiram								N.A. (>0.023 mg/kg, Thiram)									
21	Thiram	Tomato, Cucumber, Watermelon	KOH, Ethanol, Cu^{2+}, CCl_4, Water	None	UV-Vis (430 nm)	3	94.7-104.9%	0.04-1.00	N.A. (>0.04 mg/kg)	N.A.	N.A.	Iran	2013	Rastegarzadeh et al.	Department of Chemistry, College of Science, Shahid Chamran University, Ahvaz	DOI: 10.1016/j.saa.2013.05.020	Novel method for Thiram through Microextraction	N.A.

**22**	Ferbam<br>Mancozeb<br>Maneb<br>Metiram<br>Nabam<br>Propineb<br>Thiram<br>Zineb<br>Ziram	Apple, Pear, Plum, Grape, Papaya, Broccoli, Tomato	NaOH, $NaHCO_3$, DL-Penicillamine	HPLC	MS-MS	17	39-220%	0.03-2.69	11(>0.05 mg/kg)	0	1 (Broccoli)<br>2 (Plum)<br>5 (Apple, Pear, Grape)<br>7 (Papaya)	Denmark	2013	Schmidt et al.	National Food Institute, Technical University of Denmark, Soborg	DOI: 10.1080/19440049.2013.801083	The CS_2 MRLs are not referred to Zineb, Nabam, Ferbam because they are not included in the extended definition for DTCs.	EU (2010). Commission Regulation (EU) No. 750/2010 of 7 July 2010 amending annexes ii and iii to regulation (EC) No. 396/2005 of the European parliament and of the council as regards maximum residue levels for certain pesticides in or on certain products. Official Journal L 220, 21/08/2010 P. 0001–0056.
**23**	Mancozeb	Fruit Juice, Water	Dopamine Dithiocarbamate Silver NPs (DDTC-Ag NPs), TEA, Dopamine, $AgNO_3$, $NaBH_4$,	None	Vis (620 nm)<br>HNMR<br>FT-IR<br>TEM	N.A.	94.4-97.5%	N.A.	N.A.	N.A.	N.A.	India	2014	Rohit et al.	Department of Applied Chemistry, National institute of Technology, Surat	DOI: 10.1016/j.snb.2014.04.043	Use of Dopamine Dithiocarbamate functionalized silver nanoparticles	N.A.
**24**	Ethylene thiourea	Apple, Papaya, Strawberry	Methanol, NaOH, DCM, Water	HPLC	MS-MS	90	75-110%	0.001-0.0053	32 (>0.001 mg/kg, ETU)	N.A.	N.A.	Brazil	2014	Rossi Lemes et al.	Sao Paulo University, Faculty of Public Health, Department of Environmental Health, Sao Paulo	DOI: 10.1016/j.foodcont.2014.01.015	Optimization and in-house validation of a method by liquid chromatography tandem mass spectrometry	N.A.

**25**	Disulfiram	Apple, Grape, Lettuce	Gold Nanoparticles (AuNPs), Acetonitrile, Chloroform, Sulfuric Acid, KH_2PO_4, Na_2HPO_4, NaOH	UHPLC	ED	3	94.3-108.8%	0.5-15	N.A. (>0.551 mg/kg, Disulfiram)	0	N.A.	Thailand	2015	Charoenkitamorn et al.	Electrochemistry and Optical Spectroscopy Research Unit, Department of Chemistry, Faculty of Science, Chulalongkorn University	DOI: 10.1016/j.talanta.2014.09.020	Development of gold nanoparticles modified electrode for thiram, disulfiram and derivatives	N.A.
	Thiram							0.07-15	N.A. (> 0.074 mg/kg, Thiram)									
	N,N-diethyl-N',N'-dimethyl thiuram disulfide							0.07-12	N.A. (>0.076 mg/kg, DEDMTDS)									
**26**	Dazomet	Apple, Leek, Tomato, Pine needles	EDTA, NaOH, TBAHS, HCl, Water, Metal Iodide, Chloroform, Hexane, 1-2 propanediol	HPLC	UV (272 nm)	N.A.	92-100%	N.A.	N.A. (>0.0013 mg/kg)	N.A.	N.A.	Lebanon	2016	Al-Alam et al.	Azm Center for Research in Biotechnology and its Applications, Lebanese University, Tripoli	DOI: 10.1093/chromsci/bmw198	Analysis of DTCs in Vegetables using HPLC-UV followed by AAS	N.A.
	Metam Sodium								N.A. (>0.0026 mg/kg)									
	Ferbam								N.A. (>0.0017 mg/kg)									
	Ziram																	
	Zineb								N.A. (>0.0043 mg/kg)									
	Maneb				AAS													
	Mancozeb																	
	Metiram																	
	Nabam																	
	Propineb								N.A.(>0.0066 mg/kg)									
**27**	Propineb	Beer, Fruit Juice, Malt	$NaHCO_3$, Dimethyl Sulfate, NaCl, DTT, Acetonitrile	HPLC	MS-MS	24	92.2-112.6%	0.005-0.100	0 (>0.0023 mg/kg, Malt; >0.0014 mg/kg, Grape Juice; >0.0040 mg/kg, Apple Juice;	0	N.A.	Japan	2017	Kakitani et al.	Department of Analytical Technology Development, Food Safety Laboratories, Asahi Group Holdings	DOI: 10.1584/jpestics.D17-025	Rapid and sensitive analysis using modified QuEChERS method	N.A.

									>0.0052 mg/kg, Beer)									
	Mancozeb							0.005-0.100	0 (>0.0049 mg/kg, Malt; >0.0013 mg/kg, Grape Juice; >0.0050 mg/kg, Apple Juice; >0.0055 mg/kg, Beer)									
	Maneb							N.A.	N.A.									
	Zineb							N.A.	N.A.									
	Polycarbamate as EB							N.A.	N.A.									
	Milneb							N.A.	N.A.									
	Thiuram							0.05-1.5	0 (>0.0627 mg/kg, Malt; >0.0655 mg/kg, Grape Juice; >0.0489 mg/kg, Apple Juice; >0.0697 mg/kg, Beer)									
	Nickel diethyldithiocarbamate							N.A.	N.A.									
	Polycarbamate as DD							N.A.	N.A.									

	Ferbam							N.A.	N.A.									
	Ziram							N.A.	N.A.									
**28**	Propineb	Banana	HCl, $SnCl_2$, Ascorbic Acid, n-hexane, Acetonitrile, NaCl	GC	FPD	N.A.	104.2-115.4%	N.A.	N.A. (> 0.010 mg/kg)	N.A.	N.A.	China	2017	Song et al.	Institute of Pesticide & Environmental Toxicology, Guangxi Key Laboratory Cultivation Base of Agro-Environment and Agro-Product, Guangxi University, Nanning	DOI: 10.1080/03601234.2017.1399765	GC-FPD and LC-MS/MS methods for detection of DTCs in banana and soil	N.A.
	Propylene thiourea			HPLC	MS-MS		82.0-99.1%		N.A. (>0.005 mg/kg)									
	Propylene diamine						75.3-102.8%											
**29**	Thiram	Strawberry, Cucumber	DCM, AuNPs, Cellulose p-toluene sulfonates (CTSAs)	None	SERS	N.A.	91.76-112.3%	0.1-12	N.A. (>0.5 mg/kg)	N.A.	N.A.	China	2018	Chen et al.	College of Chemistry and Chemical Engineering, Central South University, Changsha, Hunan	DOI: 10.1021/acs.analchem.8b03940	Simultaneous in situ extraction and fabrication of Raman scattering Substrate for Thiram	N.A.
**30**	Ziram	Water, Tomato, Mango Beverage	AgNPs, $AgNO_3$, $NaBH_4$, Sodium Dodecyl Sulfate (SDS)	None	UV-Vis (400-570 nm)	45	94.8-108.4%	0.196-0.734	N.A. (>0.452 mg/kg)	N.A.	N.A.	Pakistan	2019	Ghoto et al.	Institute of Advanced Research Studies and Chemical Sciences, University of	DOI: 10.2116/analsci.18P417	New Colorimetric probe with Silvern Nanoparticles and SDS	N.A.
	Zineb						93.7-105.4%	0.018-0.066	N.A. (>0.012 mg/kg)									
	Maneb						93.2-107.6%	0.017-0.064	N.A. (>0.028 mg/kg)									

															Sindh, Jamshoro			
31	Ziram	Water, Tomato, Mango Beverage	CuNPs, $CuCl_2 \cdot 2H_2O$, CTAB, Hydrazine Monohydrate	None	UV-Vis (490-570 nm)	45	95.8-108.5%	0.098-0.979	N.A. (>0.458 mg/kg)	N.A.	N.A.	Pakistan	2019	Ghoto et al.	Institute of Advanced Research Studies and Chemical Sciences, University of Sindh, Jamshoro	DOI: 10.1007/s40097-019-0299-4	Copper Nanoparticles for Colorimetric detection of DTCs	N.A.
	Zineb						100.7-106.2%	0.009-0.088	N.A. (>0.002 mg/kg)									
	Maneb						96.2%-108.5%	0.008-0.085	N.A. (>0.019 mg/kg)									
32	Ziram	Apple Black Tea	AuNBPs, $AuCl_4$, CTAC, Citric Acid, $NaBH_4$, 8-Hydroxyquinoline, CTAB	None	UV-Vis (525-683 nm)	10	90-104%	0.91-1.21	N.A. (>0.91 mg/kg)	N.A.	N.A.	China	2020	Wang et al.	State Key Laboratory of Ecological Pest Control for Fujian and Taiwan Crops, College of Plant Protection, Fuzhou	DOI: 10.1016/j.aca.2020.09.032	Visual Screening of Total DTCs in foods using gold nanopyramids	N.A.
	Thiram							0.72-0.96	N.A. (>0.72 mg/kg)									
	Zineb							0.82-1.09	N.A. (>0.82 mg/kg)									
33	Propineb	Infant Formula, Black tea	EDTA, $K_2S_2O_8$, K_2CO_3, NaOH, Acetone, Methanol, DCM, Acetonitrile	GC	MS	N.A.	98-103%	1.05-9.77	N.A. (>0.52 mg/kg)	N.A.	N.A.	Turkey	2020	Bodur et al.	Yildiz Technical University, Faculty of Art and Science, Department of Chemistry, Davutpasa, Esenler, Istanbul	DOI: 10.1016/j.talanta.2020.120846	Dispersive liquid-liquid microextraction followed by GC-MS	N.A.
34	Ziram	Apple Juice	Ethanol, Water, NaBr, AgNPs	None	SERS	N.A.	N.A.	N.A.	N.A. (>12.6 x 10^{-8} mol/L)	N.A.	N.A.	China	2020	Wei et al.	College of Materials Science and Engineering, Beijing	DOI: 10.1039/d0ay01953d	Surface-enhanced Raman spectroscopy combined with an	N.A.
	Thiram								N.A. (>9.9 x									

									10 -7 mol/L)						University of Chemical Technology, Beijing		exhaustive peak-seeking method	
**35**	Thiram<br>Mancozeb<br>Propineb	Lettuce, Broccoli	AgAu Nanopillars, Water, AgNPs	None	SERS	N.A.	65-134%	0.5-7	N.A.	N.A.	N.A.	Taiwan	2021	Tsen et al.	Department of Biomedical Engineering and Environmental Sciences, National Tsing Hua University	DOI: 10.1016/j.apsusc.2021.149740	Surface-enhanced Raman spectroscopy	N.A.
**36**	Thiram	Tap Water, Orange juice	e-AuNPs, Sodium Citrate, NH_4OH Ascorbic Acid	None	SERS	N.A.	68-114%	N.A.	N.A. (>1.7 x 10 -9 mol/L)	N.A.	N.A.	Vietnam	2022	Ahn et al.	Phenikaa Unicrsity Nano Institute (PHENA), Hanoi	DOI: 10.1016/j.apsusc.2022.152555	Raman spectroscopy Nanosensors	N.A.
**37**	Mancozeb	Chamomile	Dimethyl Sulfate, Acetonitrile, Formic Acid, Ethyl acetate, L-Cysteine, EDTA-Na_2	HPLC	MS-MS	N.A.	67.5-109%	0.01-0.1	N.A. (>0.05 mg/kg)	N.A.	N.A.	Egypt	2022	Sayed et al.	Agricultural Research Center, Central Laboratory of Residue Analysis of Pesticides and Heavy Metals in Foods, Giza	DOI: 10.1016/j.jfca.2022.104646	Method with modified QuEChERS	N.A.
**38**	Ethylene thiourea<br>Propylene thiourea	Grape, Cherry Tomato, Strawberry	Water, Methanol	HPLC	ICP-MS	N.A.	87-101%<br>98-99%	0.01-1.00	N.A. (>0.022 mg/kg)<br>N.A. (>0.010 mg/kg)	N.A.	N.A.	France	2023	Bendhiab et al.	Laboratory for Food Safety, French Agency for Food, Environm	DOI: 10.1007/s00216-023-05034-6	Liquid chromatography hyphenated to inductively coupled	N.A.

															ental and Occupational Health & Safety (ANSES), University of Paris Est		plasma-tandem mass spectrometry	
**39**	Thiram	Water, Apple, Guava, Broad Beans, Green Beans	Si-AgNPs, Water	None	UV-Vis (420 nm)	N.A.	N.A.	N.A.	N.A. (>0.15 mg/kg)	N.A.	N.A.	India	2023	Eswaran et al.	Department of Chemistry, B.S. Abdure Rahman Crescent Institute of Science and Technology, Vandalur	DOI: 10.1016/j.jfca.2023.105327	Use of silane-modified silver nanoparticles	N.A.
**40**	Ethylene thiourea	Cucumber, Celery, Tomato, Green pepper, Potato, Citrus, Apple, Jujube, Raisins, Grape	Water, methanol, NaCl, $MgSO_4$	None	Fluorescence (450-570 nm)	10	95.04-105.41	0.25-10	N.A. (>0.74 mg/kg)	N.A.	N.A.	China	2023	Han et al.	College of Science, Sichuan Agricultural University	DOI: 10.1016/j.microc.2023.108549	Ratiometric fluorescence determination of ethylene thiourea	N.A.
**41**	Maneb	Tomato, Rice, Papaya	EDTA, Water, Triton X-114, DMSO, CTAB, DTAB, DDAB, SDS; AIT, SB3-10, Cu(II) solution	None	Digital Images	N.A.	71-105%	0.265-33.15	N.A. (>1.00 mmol/L)	N.A.	N.A.	Brazil	2023	Martins et al.	Center for Nuclear Energy in Agriculture, University of Sao Paulo	DOI: 10.1016/j.microc.2025.113614	Microextraction coupled with digital images colorimetric detection	N.A.
**42**	Ziram	Green tea, Flower tea, Red tea, Black tea	L-Cysteine, CH_3I, TBAHS, Na_2EDTA, Citric Acid, L-	HPLC	CDCL	N.A.	85-116.45 %	0.0005-0.140	N.A. (>0.00054 mg/kg)	N.A.	N.A.	China	2024	Wei et al.	State Key Laboratory of Food Science and Resources ,	DOI: 10.1016/j.foodchem.2024.140237	Novel HPLC-CDCL method utilizing nitrogen-doped	N.A.
	Zineb						74.51-104.38 %	0.0023-0.280	N.A. (>0.0023 mg/kg)									

	Propineb		Serine, $Ce_2SO_4 \cdot 4H_2O$, TFA, MSA				73.21-101.71%	0.005-0.450	N.A. (>0.005 mg/kg)						Nanchang University		carbon dots for sensitive and selective detection	
**43**	Zineb	Green Tea, White Tea, Black Tea	$Mn(CH_3COOH)_3 \cdot 2H_2O$, TCPP, Tyrosine, 4-APP	None	Fluorescence (500-650 nm)<br>UV (365 nm)	N.A.	89.5-102.5%	0.01-0.2	N.A.	N.A.	0.1 (Tea)	China	2025	Feng et al.	State Key Laboratory of Tea Plant Biology and Utilization, Anhui Provincial Key Laboratory of Food Safety Monitoring and Quality Control, Hefei	DOI: 10.1016/j.snb.2024.137026	Colorimetric and Fluorescent double-mode probe for DTCs detection	N.A.
